# Development and biomarker-based validation of a novel feed formulation for managing production diseases in dairy buffaloes

**DOI:** 10.3389/fvets.2026.1937866

**Published:** 2026-09-08

**Authors:** Savleen Kour, Neelesh Sharma, Sung-Jin Lee

**Affiliations:** 1 Division of Veterinary Medicine, Faculty of Veterinary Science & Animal Husbandry, Sher-e-Kashmir University of Agricultural Sciences & Technology of Jammu (SKUAST-J), Ranbir Singh Pura, Jammu, Jammu and Kashmir, India; 2 Department of Applied Animal Science, College of Animal Life Sciences, Kangwon National University, Chuncheon, Republic of Korea

**Keywords:** cytokines, gene expression, hypocalcemia, negative energy, qPCR, transition feed

## Abstract

**Introduction:**

The critical period from 3 weeks prepartum to 3 weeks postpartum is considered the transition period and is characterized by major endocrinological and metabolic changes that can lead to production and infectious diseases. In addition, decreased dry matter intake and altered nutritional requirements are evident during this period. The objective of our study was to analyze the effects of transition feed fed to buffaloes on major hematobiochemical parameters, oxidative stress, cytokine levels, and mRNA expression of molecular markers associated with production diseases.

**Methods:**

A total of 20 buffaloes were selected, irrespective of age and parity, and divided into two groups: a transition feed-supplemented group (Group I, *n* = 10) and a control group (Group II, *n* = 10). Proximate analysis of the transition feed was performed using the Weende method. Parameters such as body condition score, milk production, urine pH, hematobiochemical parameters, and oxidative stress markers were analyzed according to their respective optimized standard protocols. β-Hydroxybutyric acid (BHBA), tumor necrosis factor-α (TNF-α), and interferon-γ (INF-γ) were evaluated using ELISA kits. Genes selected for hypocalcemia (SNF1-like kinase [NUAK1] and neuroendocrine secretory protein 55 [NESP55]), negative energy balance (carnitine palmitoyl transferase 1A [CPT1A] and insulin-like growth factor-1 [IGF-1]), and cytokines were evaluated using quantitative real-time polymerase chain reaction (qPCR).

**Results:**

Weende’s analysis showed an improved chemical composition of the transition feed, while significant improvements in urine pH, white blood cell (WBC) counts, lymphocyte counts, red blood cell (RBC) counts, total protein, albumin, and antioxidant defense enzymes, including superoxide dismutase, catalase, and glutathione peroxidase, were observed in Group I. The qPCR results showed significant upregulation of NUAK1 and CPT1A and downregulation of NESP55, IGF-1, and cytokines in the transition feed group compared with the control group. This indicates a decreased likelihood of hypocalcemia and negative energy balance in buffaloes in Group I.

**Discussion:**

This study elucidates the importance of transition feed in buffaloes, providing valuable insights into improving health and preventing infectious and production diseases. Nutritional intervention during the transition period led to significant improvements in WBC count, granulocyte count, RBC count, hemoglobin (Hb), total protein, albumin, glucose, calcium, and sodium levels in the transition feed group.

## 1 Introduction

The transition period is described as the period from 3 weeks prepartum to 3 weeks postpartum (1). This is the most critical period for dairy cattle and buffaloes because decreased milk production and high culling rates affect the economic wellbeing of farmers (2). During the critical prepartum period, feed intake is at its lowest point in the lactation–gestation cycle. This period is characterized by negative energy balance (NEB), fat mobilization, and elevated concentrations of circulating non-esterified fatty acids and ketone bodies. Major metabolic transitions occur in a dairy animal during this period, as cattle and buffaloes transition from a non-lactating to a lactating state and undergo the stress of parturition (3). Metabolic, endocrine, and immune changes occur, particularly in mammary and uterine tissues, which further recruit components of the innate immune system, including leukocytes, macrophages, and dendritic cells (4, 5). Changes in body condition score occur throughout the lactation cycle and correspond to fluctuations in energy balance of the animal (6). The nutritional requirements of the fetus reach their maximum level during the 3 weeks prepartum, but dry matter intake decreases by 10–30% (7). There is an increase in milk yield, milk protein, and fat within 3 weeks of the onset of lactation, which rapidly exceeds feed intake (8). Feeding during the transition period determines cattle productivity during the subsequent lactation period. The diet of dairy cattle changes sharply from forage-based to concentrate-rich at calving (8). The energy required for milk production during peak lactation often exceeds the available energy. As a result, body lipid reserves are mobilized to compensate for the energy deficit and meet the demands of milk production. Therefore, animals begin to lose body condition. A reduction in net energy intake relative to energy expenditure results in NEB (9). Adipose tissue is considered a source of several pro-inflammatory cytokines, such as interleukins and tumor necrosis factor-α (TNF-α). Enhanced expression of these cytokines can induce a pro-inflammatory environment and facilitate oxidative damage, including increased free radical production (10). Increased hepatic triglyceride accumulation (fatty liver) and increased expression of metabolic genes related to fatty acid uptake and storage have been observed following administration of recombinant bovine TNF-α during late pregnancy in cattle. Conversely, decreased expression of genes involved in processes such as gluconeogenesis suggests reduced glucose production associated with inflammatory pathways (11). Numerous studies have shown a relationship between high body condition score (BCS), greater BCS losses, and drastic changes in metabolic activity, leading to increased oxidative stress in transition cows (12, 13). Providing appropriate nutrition during this period greatly improves calving ease, cow and calf welfare, milk production, and reproductive performance (14). There is limited information on how nutritional management influences physiological adaptation in buffaloes. In India, buffaloes constitute an important component of dairy production and may exhibit responses that cannot be directly extrapolated from dairy cattle. In view of these considerations, our study aimed to analyze the impact of nutritional intervention on the occurrence of metabolic disturbances in terms of hematobiochemical parameters, oxidative stress indicators, cytokine responses associated with inflammation, and molecular markers during the transition period in buffaloes. This study provides a multidimensional approach to integrating nutrition and management strategies for buffaloes during the transition period to facilitate a smooth onset of lactation.

## 2 Materials and methods

### 2.1 Animals and feeding management

To determine the impact of nutritional interventions in buffaloes during this critical period, a transition feed was formulated to assess its potential effects on overall metabolic performance and productivity. In total, 17 ingredients were used in this formulation. Ingredients such as crushed oats, maize, rice, and rice bran were selected as energy sources, whereas meals and cakes were used as protein sources. The crushed maize and oats and de-oiled bran were pulverized to an appropriate size, and the pelletized cottonseed cake and formaldehyde-treated mustard oil cake produced after oil extraction were reduced in size for easy incorporation. The soyabean meal was obtained as a coproduct of soyabean oil extraction and subsequently heat-treated to inactivate antinutritional factors. Ingredients such as mineral mixture, salt, limestone powder, vitamins A and D3, vitamin E (Sigma-Aldrich Company), magnesium sulfate, magnesium oxide, dicalcium phosphate, and sodium bicarbonate were incorporated without antibiotics. The composition contained fixed percentages of ingredients provided to buffaloes during the transition period. No additional treatments were applied to any ingredients used in the composition. Manual mixing of ingredients was initiated by incorporating small proportions of wet ingredients (vitamins A, D, and E) gradually into dry carriers such as bran, oats, and mineral mixture to form a premix. The meals and cakes were then gradually and uniformly incorporated into the premix to avoid lump formation. The formulated feed for the transition period was evaluated for proximate composition (Weende analysis), including moisture, total ash, crude protein, ether extract, and crude fiber.

Before the start of the experiment, all 20 buffaloes were stratified based on parity (mean ± SD, 3.2 ± 0.8), age (6.5 ± 1.2 years), and body weight (545 ± 38 kg). Using a computer-generated random number sequence, the animals were then randomly allocated to either the transition feed-supplemented group (Group I, n = 10) or the control group (Group II, n = 10), ensuring that both groups were balanced for these parameters (*p* > 0.05 between groups for all baseline characteristics). BCS was also assessed at −30 days relative to calving, and no significant differences were observed between the groups at baseline (Group I: 3.2 ± 0.4; Group II: 3.3 ± 0.3; *p* = 0.54) (Supplementary Table 1). The study was conducted over a 2-month period in the R.S. Pura area of the Jammu region, UT J&K, India.

To ensure dietary consistency and minimize variation, both Group I and Group II were managed under a controlled feeding regimen. The quantity of basal roughages (e.g., wheat straw, green fodder) offered to both groups was standardized based on the average body weight of the animals at the beginning of the trial (offered at 2.5% of body weight on a dry matter basis and adjusted weekly). The daily concentrate allowance for the control group (Group II) was fixed at 2.5 kg per animal per day throughout the transition period, rather than being varied based on milk production or fodder availability, to avoid confounding the comparison with the supplemented group. The transition feed supplement offered to Group I (1 kg/day) replaced an equivalent amount of the control concentrate to maintain isocaloric and isonitrogenous conditions as closely as possible. All animals were housed in individual stalls during the feeding periods to ensure that each animal consumed its allocated ration (see Table 1).

**Table 1:** Ingredient composition of the transition feed supplement (group I) and control concentrate feed (group II).

S No.	Feed supplement (group I)		Control concentrate feed (group II)	
	Feed ingredients	Parts (kg)	Feed ingredients	Parts (kg)
1	Crushed maize	12	Crushed maize	16
2	Crushed oats	10	Crushed barley	9
3	Rice	7	Cattle pelleted feed, type II	13
4	Wheat bran	10	Wheat bran	18
5	De-oiled bran	10	Mustard oilcake	20
6	Rice bran	13	Cottonseed cake	23
7	Soybean meal	11.5	Salt	1
8	Formaldehyde-treated mustard oil cake	12.1		
9	Cottonseed cake	10		
10	Mineral mixture	1		
11	Salt (common salt)	1		
12	Limestone powder	1		
13	Dicalcium phosphate	0.65		
14	Sodium bicarbonate	0.35		
15	Magnesium oxide	0.25		
16	Magnesium sulfate	0.12		
17	Vitamins A, D3, and E	0.03		
	Total	100 parts	Total	100 parts

### 2.2 Sampling protocol and blood collection for parameter evaluation

Blood samples were collected from a selected group of buffaloes via the jugular vein at predetermined time points (−30, +15, 0, +15, and +30 days) during the transition period. Blood samples were collected in vacutainers containing EDTA (ethylenediaminetetraacetic acid) for the analysis of hematologic parameters using the MYTHIC 18 VET Hematology Analyzer (Compact Diagnostics (India) Pvt. Ltd.). Similarly, blood samples collected in tubes containing serum clot activators were used to estimate cytokines (interferon-γ and tumor necrosis factor-α) and biochemical parameters. Serum was separated by centrifugation at 3,000 rpm for 10 min and stored at −20 °C. Biochemical parameters such as total protein, albumin, cholesterol, bilirubin, and blood urea nitrogen (BUN) were estimated by UV spectrophotometry using Erba diagnostic kits (Transasia Biomedical Ltd., Mumbai, India). Blood electrolytes, including sodium and potassium, were measured using Liquizyme kits (Beacon Diagnostics Pvt. Ltd., Navsari, India), and magnesium and inorganic phosphorus were measured using Agappe diagnostic kits (Ernakulam, Kerala, India). Heparin-containing blood samples were used to estimate oxidative biomarkers, including glutathione peroxidase (GPX), lipid peroxidation (LPO), superoxide dismutase (SOD), and catalase. Blood glucose levels were estimated using an Accu-Chek glucometer immediately after blood collection from the ear vein. Total RNA was extracted from EDTA-preserved whole blood using the TRIzol method (Invitrogen Life Technologies, Carlsbad, CA, United States). Extracted RNA was used for cDNA synthesis, followed by quantitative real-time polymerase chain reaction (qRT-PCR), to determine the expression of genes specific to production diseases during the transition period. The molecular genes selected for milk fever/hypocalcemia were SNF1-like kinase (NUAK1) and neuroendocrine secretory protein 55 (NESP55); for ketosis/NEB, carnitine palmitoyl transferase 1A (CPT1A) and insulin-like growth factor 1 (IGF-1); and for cytokine determination, tumor necrosis factor-α (TNF-α) and interferon-γ (IFN-γ) were selected. The primer sequences, annealing temperatures, and qPCR conditions used are depicted in Supplementary Table 1.

qRT-PCR was conducted using a qRT-PCR cycler, MyGo Mini (MyGo 002). A reaction mixture was prepared containing 10 μL of HiMedia Hi-SYBr Master Mix (Cat. No. MBT074-100R, HiMedia, Mumbai, India), 0.3–0.5 μL each of the forward and reverse primers, 2 μL of cDNA, and nuclease-free water to a final volume of 20 μL. The cycling conditions for qPCR are provided in Supplementary Table 2.

### 2.3 Body condition score

**Figure 1:**
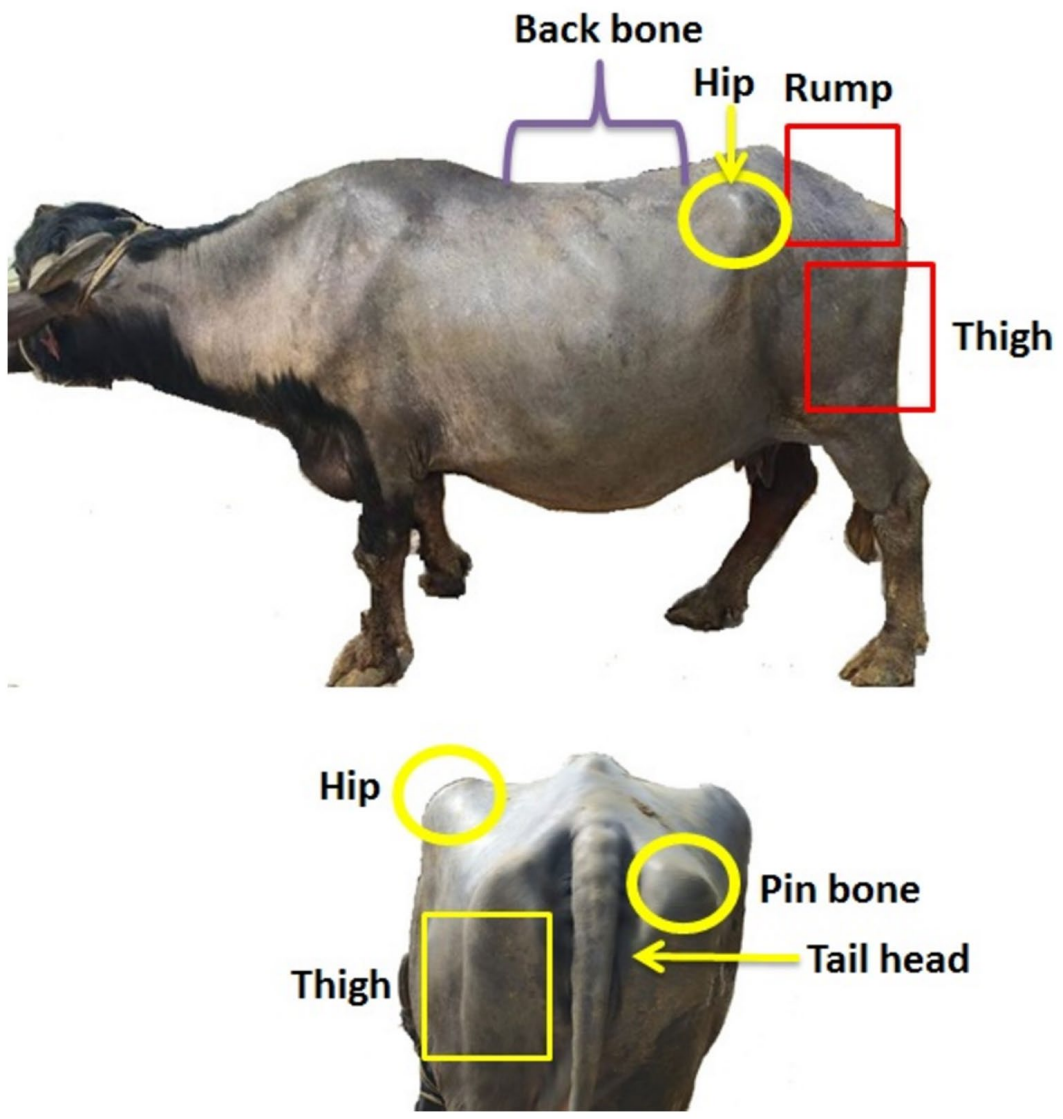
Measurement of body condition score in buffaloes.

Buffaloes were scored based on the appearance and palpation of the back and hindquarters according to Wildman et al. (15). Factors considered were the thoracic and lumbar regions of the vertebral column (chine, loin, and rump), spinous processes (loin), anterior coccygeal vertebrae (tailhead), tuber sacrale (hooks), and tuber ischii (pin bones) (Figure 1). Body condition was scored on a scale of 1 to 5 based on the factors described above.

### 2.4 Milk samples

Milk samples from postpartum buffaloes were tested on-site using the Modified California Mastitis Test (MCMT). After proper disinfection of the teat surface with 70% ethyl alcohol, a few streams of milk were collected from each quarter. Milk was collected in sterile, screw-capped, wide-mouth polyethylene vials. The samples were kept in an ice box and transported to the laboratory for further processing. Milk samples were tested for somatic cell count by using a Cell Counter DCC (DeLaval, Sweden). Previous-lactation milk yield was recorded through a farmer-reported questionnaire, and current milk yield was assessed during the 7th to 8th week postpartum.

### 2.5 Urine samples

Urine samples from animals in both Group I and Group II were collected for pH determination as an indicator of the acidifying capacity of the ration during the transition period (−30, −15, 0, +15, and +30 days).

### 2.6 Oxidative stress markers

For evaluation of superoxide dismutase (SOD), glutathione peroxidase (GPx), lipid peroxidase (LPO), and catalase, 5 mL of blood was collected in a heparin-containing vacutainer and centrifuged at 3,000 rpm for 10 min. Plasma was then stored at −20 °C, and the RBC lysate was used to determine the antioxidant status. The RBC lysate was repeatedly washed with normal saline in a 1:1 ratio and then centrifuged for 10 min at 3,000 rpm. A 1% lysate (100 μL of lysate and 990 μL of distilled water) and a 33% lysate (330 μL of lysate and 640 μL of PBS, pH 7.4) were prepared. For this experiment, the 1% RBC lysate was used. Lipid peroxidation in erythrocytes was determined by evaluating malondialdehyde production according to the method described by Rehman (16). Plasma catalase activity was determined according to the method described by Aebi (17). Erythrocytic superoxide dismutase activity and glutathione peroxidase activity were determined according to the methods described by Marklund and Marklund (18, 19), respectively.

### 2.7 Estimation of β-hydroxybutyric acid, bovine tumor necrosis factor-α, and interferon-γ using ELISA kits

Determination of β-hydroxybutyric acid (BHBA) concentration (nmol/ml) from collected serum was performed using an ELISA kit (Immunotag; GBiosciences, United States) following the manufacturer’s standard protocol supplied with the kit. Similarly, TNF-α (ng/ml) and INF-γ (ng/ml) were estimated from stored serum samples using ELISA kits (RayBio, Georgia, United States). All reagents were brought to room temperature [18–25 °C] before use, and final concentrations were prepared according to the directions provided in the manual. The optical density (OD) values of the standards [X-axis] were plotted against their known concentrations to generate standard curves [Y-axis], from which the TNF-α and INF-γ concentrations in the samples were determined.

### 2.8 Statistical analysis

All statistical analyses were performed using IBM SPSS Statistics for Windows (Version 26.0; IBM Corp., Armonk, NY, United States) and GraphPad Prism (Version 9.0; GraphPad Software, San Diego, CA, United States). The normality of residuals and homogeneity of variances were assessed using the Shapiro–Wilk test and Levene’s test, respectively. Variables that did not meet the parametric assumptions were log- or square-root-transformed before analysis.

### 2.9 Linear mixed-effects model for repeated measures

Given that repeated measurements were collected from the same animals across multiple time points, the data were analyzed using a linear mixed-effects model (LMM) with a repeated-measures covariance structure. This approach accounts for the inherent correlation between successive observations within the same animal and handles missing data more robustly than traditional analysis of variance (ANOVA).


$$Y_{\mathrm{igt}}=\mu +\alpha_{g}+\beta_{t}+{\left(\alpha \beta \right)}_{\mathrm{gt}}+\gamma_{i}+\varepsilon_{\mathrm{igt}}$$

**Table:** 

Component	Description
*Y_igt_*	Observed value of the dependent variable (e.g., BHBA, TNF-α, SOD, or gene expression) for buffalo *i* in group *g* at time *t*
*μ*	Overall grand mean (intercept)
*α_g_*	Fixed effect of treatment group (*g* = Group I or Group II)
*β_t_*	Fixed effect of time (*t* = −30, −15, 0, +15, or +30 days relative to calving)
*(αβ)_gt_*	Fixed interaction effect between group and time (tests whether the trajectory over time differs between the two groups)
*γ_i_*	Random effect of the individual animal (buffalo ID nested within group) → accounts for baseline interanimal variability
*ε_igt_*	Residual error term

The Ct values of the GAPDH gene for the control and experimental groups were analyzed, and statistical significance was assessed using an independent-samples t-test. Statistical analysis was performed on the ΔΔCt values using an independent-samples t-test, and the results were then converted to 2^−ΔΔCt^ for data presentation.

#### 2.9.1 Ethical approval/consent

Ethical approval was granted for the experiment under approval no. V-11011(13)/4/2025-CPCSEA-DAHD.

## 3 Results

### 3.1 Proximate composition of transition feed

The formulated feed for the transition period was evaluated for the proximate composition (Weende analysis), including moisture content, total ash, crude protein, ether extract, and crude fiber. The estimated compositions of the concentrate feed fed to the transition feed-supplemented group (Group I) and the control group (Group II) are presented in Tables 2, 3, respectively.

**Table 2:** Chemical composition of the transition feed supplement.

S no.	Chemical composition	Percentage
1	Dry matter	91.50
2	Organic matter	88.74
3	Crude protein	20.08
4	Ether extract	3.72
5	Crude fiber	17.00
6	Total ash	11.85

**Table 3:** Chemical composition of the control concentrate feed.

S no.	Chemical composition	Percentage
1	Dry matter	90.79
2	Organic matter	88.74
3	Crude protein	20.58
4	Ether extract	3.38
5	Crude fiber	15.52
6	Total ash	10.26

### 3.2 Effect of supplemented feed on body condition score

In this study, BCS was evaluated on a scale of 1 to 5, in 0.5-point increments. The BCS values showed no significant differences (*p* < 0.05) between the transition feed group (Group I) and the control group (Group II). However, a significant (*p* < 0.05) decrease in the BCS from −30 to 0 day in Group I and from 0 to +30 days in Group II was observed (Table 4).

**Table 4:** Profile of body condition scores at different time points during the transition period in buffaloes.

BCS	Periods			GM
	−30 days	0 day	+30 days	
Group I	3.25^a^ ± 0.11	3.00^b^ ± 0.07	2.90^b^ ± 0.08	2.98^A^ ± 0.07
Group II	3.10^a^ ± 0.07	3.00^a^ ± 0.02	2.55^b^ ± 0.05	2.88^A^ ± 0.05
PM	3.18^a^ ± 0.07	3.00^b^ ± 0.04	2.83^c^ ± 0.05	

^A, B&a,b,c^ Means with different superscripts between columns and rows differ significantly (*p* < 0.05). PM and GM denote period mean and group mean, respectively.

For BCS, the LMM with an AR(1) covariance structure revealed a significant main effect of Time (F(2, 36) = 16.84, *p* < 0.001), indicating a significant decline in BCS across the transition period. A significant main effect of Group was also observed (F(1, 18) = 6.23, *p* = 0.022), with Group I (overall mean: 2.98 ± 0.07) maintaining a higher BCS than Group II (overall mean: 2.88 ± 0.05). Importantly, the Group × Time interaction was significant (F(2, 36) = 4.67, *p* = 0.016), demonstrating that the pattern of BCS change over time differed between the supplemented and control groups.

*Post hoc* Bonferroni-adjusted pairwise comparisons revealed that Group I had significantly higher BCS than Group II at +30 days (mean difference = 0.35, 95% CI: 0.21 to 0.49, *p* < 0.001). No significant differences were observed between groups at −30 days (mean difference = 0.15, 95% CI: −0.07 to 0.37, *p* = 0.168) or at 0 day (mean difference = 0.00, *p* = 1.000).

Within Group II, BCS declined significantly from −30 to +30 days (mean difference = −0.55, 95% CI: −0.72 to −0.38, *p* < 0.001). In Group I, the decline from −30 to +30 days was also significant but less pronounced (mean difference = −0.35, 95% CI: −0.58 to −0.12, *p* = 0.006). These findings indicate that transition feed supplementation attenuated the loss of body condition during the periparturient period.

### 3.3 Effect of transition feed on the milk production and occurrence of mastitis

The milk yield improved from 6.0 kg/day before supplementation to 8.35 kg/day after supplementation with transition feed in Group I, representing an increase of 2.35 kg/day and 39.16% of yield. The transition feed supplementation resulted in a substantial improvement in milk production, with a large paired effect size (Cohen’s dz. = 3.21). The mean increase in milk yield was estimated to be 1.79–2.83 kg/day based on the 95% confidence interval (n = 10). In Group II, average milk yield increased from 6.00 to 6.25 kg/day, representing a 4.17% improvement (0.25 kg/day). The control group showed a mod erate-to-large within-animal effect, with a paired Cohen’s d_2_ of 0.81, with a 95% confidence interval for the mean increase ranging from 0.03 to 0.57 for 10 buffaloes. Milk production was significantly higher (*p* < 0.001) in Group I (8.35 ± 0.18 kg/day) than in Group II (6.25 ± 0.20 kg/day), representing a 33.6% increase. Analysis of the milk samples revealed that a higher number of animals in the control group (*n* = 4) were positive for mastitis by the MCMT (T/+) compared with the feed group (*n* = 2), with average somatic cell counts of 284 ± 14.8 (×10^3^/ml) and 264 ± 7.08 (×10^3^/ml), respectively. However, the MCMT score T/+ was negligible.

### 3.4 Effect of transition feed/supplemented feed on urine pH

Urine pH in Group I declined progressively from 8.25 ± 0.12 at −30 days to 6.95 ± 0.08 at 0 day and 6.60 ± 0.10 at +15 days, indicating effective acidification of the urine. In contrast, Group II showed only a modest decline from 8.40 ± 0.10 to 7.80 ± 0.09 at 0 day and 7.70 ± 0.08 at +15 days. The difference in urine pH between groups was significant at 0 day (mean difference = −0.85, *p* < 0.001), +15 days (mean difference = −1.10, *p* < 0.001), and +30 days (mean difference = −1.00, *p* < 0.001)”.

### 3.5 Effect of transition feed on hematological parameters

Parameters such as WBCs (x10^3^/μL), lymphocytes (%), granulocytes (%), RBCs (x10^6^/μL), hemoglobin (g/dl), hematocrit (%), and blood glucose (mg/dl) showed significant differences (*p* < 0.05) between the transition feed trial group (Group I) and control group (Group II) (Table 5). In contrast, no significant differences (*p* < 0.05) were observed in the mean ± SE values of monocytes (%), MCH (mean corpuscular hemoglobin; pg), MCHC (mean corpuscular hemoglobin concentration; pg), and platelets (x10^3^/μL) among the feed trial group (Group I) and the control group (Group II) (Table 5).

**Table 5:** Mean ± SE values of hematological parameters in the transition feed and control groups at different time points during the study period.

Groups	Transition period (days)					GM
	−30	−15	0	+15	+30	
WBC						
Group I	6.49^a^ ± 0.03	6.55^a^ ± 0.04	7.34^b^ ± 0.08	7.55^c^ ± 0.13	7.73^d^ ± 0.21	7.13^A^ ± 0.09
Group II	6.42^a^ ± 0.02	6.44^a^ ± 0.10	7.09^b^ ± 0.11	6.98^ba^ ± 0.07	7.63^c^ ± 0.23	6.91^B^ ± 0.08
PM	6.46^a^ ± 0.02	6.50^a^ ± 0.05	7.21^b^ ± 0.07	7.27^ca^ ± 0.10	7.68^c^ ± 0.15	
Lymphocytes						
Group I	45.10^a^ ± 0.49	46.32^b^ ± 0.53	48.47^c^ ± 0.45	46.37^d^ ± 0.51	47.18^da^ ± 0.42	46.6A^A^ ± 0.26
Group II	43.56^a^ ± 0.28	43.88^a^ ± 0.35	47.31^b^ ± 0.33	46.31^c^ ± 0.48	46.64^ca^ ± 0.39	45.54^B^ ± 0.27
PM	44.33^a^ ± 0.33	45.10^a^ ± 0.42	47.89^b^ ± 0.30	46.34^c^ ± 0.34	46.91^ca^ ± 0.29	
Monocytes						
Group I	4.60^a^ ± 0.08	4.74^b^ ± 0.09	4.02^c^ ± 0.06	4.44^d^ ± 0.04	4.43^d^ ± 0.04	4.45^A^ ± 0.04
Group II	4.60^a^ ± 0.08	4.55^a^ ± 0.10	4.16^b^ ± 0.05	4.37^c^ ± 0.06	4.40^ca^ ± 0.05	4.42^A^ ± 0.04
PM	4.60^a^ ± 0.06	4.64^a^ ± 0.07	4.09^b^ ± 0.04	4.41^c^ ± 0.04	4.42^ca^ ± 0.03	
Granulocytes						
Group I	42.68^a^ ± 0.39	44.01^b^ ± 0.49	47.75^c^ ± 0.91	44.10^d^ ± 0.37	44.73^e^ ± 0.36	44.65^A^ ± 0.34
Group II	42.09^a^ ± 0.24	43.10^b^ ± 0.48	45.84^c^ ± 0.33	42.50^d^ ± 0.34	43.86^e^ ± 0.27	43.48^B^ ± 0.24
PM	42.39^a^ ± 0.23	43.56^b^ ± 0.35	46.79^c^ ± 0.52	43.30^d^ ± 0.30	44.30^e^ ± 0.24	
RBC (x10^6^/μL)						
Group I	5.14^a^ ± 0.11	5.25^b^ ± 0.12	5.10^c^ ± 0.08	5.25^d^ ± 0.11	5.26^d^ ± 0.11	5.20^A^ ± 0.05
Group II	5.00 ± 0.05	5.01 ± 0.12	5.08 ± 0.07	5.07^a^ ± 0.06	5.18^ba^ ± 0.07	5.07^B^ ± 0.03
PM	5.07^a^ ± 0.06	5.13^b^ ± 0.09	5.09^b^ ± 0.05	5.16^ba^ ± 0.07	5.22^c^ ± 0.07	
Hb						
Group I	10.70^a^ ± 0.35	10.83^a^ ± 0.41	9.83^b^ ± 0.18	9.46^ca^ ± 0.14	9.42^ca^ ± 0.20	10.05^A^ ± 0.15
Group II	10.04^a^ ± 0.20	10.11^a^ ± 0.14	9.84^b^ ± 0.11	9.30^c^ ± 0.17	9.28^d^ ± 0.16	9.71^B^ ± 0.08
PM	10.37^a^ ± 0.21	10.47^b^ ± 0.23	9.83^c^ ± 0.10	9.38^d^ ± 0.11	9.35^d^ ± 0.12	
HCT						
Group I	33.29^a^ ± 0.48	34.53^b^ ± 0.45	32.18^c^ ± 0.35	32.08^c^ ± 0.25	31.49^d^ ± 0.66	32.71^A^ ± 0.25
Group II	32.86^a^ ± 0.56	32.39^a^ ± 0.36	31.87^a^ ± 0.53	31.02^b^ ± 0.46	31.20^ba^ ± 0.58	31.87^B^ ± 0.24
PM	33.07^a^ ± 0.36	33.46^a^ ± 0.37	32.02^b^ ± 0.31	31.55^ba^ ± 0.28	31.34^b^ ± 0.43	
MCV						
Group I	53.87^a^ ± 0.80	55.54^b^ ± 0.70	57.72^c^ ± 0.30	57.43^c^ ± 0.31	57.44^c^ ± 0.24	56.40^A^ ± 0.31
Group II	51.87^a^ ± 0.80	53.99^a^ ± 0.49	57.54^b^ ± 0.33	57.50^ba^ ± 0.27	56.61^c^ ± 0.21	55.90^A^ ± 0.31
PM	53.87^a^ ± 0.55	54.76^b^ ± 0.45	57.63^ca^ ± 0.22	57.46^c^ ± 0.20	57.02^c^ ± 0.18	
MCH						
Group I	15.51^a^ ± 0.64	16.33^b^ ± 0.62	18.06^c^ ± 0.30	17.92^c^ ± 0.26	17.87^c^ ± 0.17	17.14^A^ ± 0.24
Group II	15.19^a^ ± 0.51	15.30^a^ ± 0.49	17.99^ba^ ± 0.29	17.69^b^ ± 0.27	17.41^b^ ± 0.11	16.72^A^ ± 0.23
PM	15.35^a^ ± 0.40	15.82^a^ ± 0.40	18.03^ba^ ± 0.20	17.81^b^ ± 0.18	17.64^b^ ± 0.11	
MCHC						
Group I	32.70^a^ ± 0.23	33.56^a^ ± 0.22	32.70^a^ ± 0.21	33.24^a^ ± 0.29	32.98^a^ ± 0.29	33.04^A^ ± 0.12
Group II	31.48^a^ ± 0.23	32.46^a^ ± 0.53	30.26^b^ ± 2.99	32.12^c^ ± 0.18	32.34^c^ ± 0.28	31.98^B^ ± 0.60
PM	32.70^a^ ± 0.16	33.01^a^ ± 0.31	31.48^b^ ± 1.49	32.68^c^ ± 0.21	32.66^c^ ± 0.21	
RDW						
Group I	17.49^a^ ± 0.41	16.84^b^ ± 0.39	14.59^c^ ± 0.23	15.81^d^ ± 0.29	16.56^e^ ± 0.22	16.26^A^ ± 0.20
Group II	18.67^a^ ± 0.41	17.35^a^ ± 0.56	15.86^b^ ± 0.23	16.47^c^ ± 0.29	16.65^ca^ ± 0.25	16.76^B^ ± 0.18
PM	17.49^a^ ± 0.28	17.09^b^ ± 0.34	15.23^c^ ± 0.21	16.14^d^ ± 0.21	16.61^d^ ± 0.16	
PLT						
Group I	353.80^a^ ± 12.74	356.30^a^ ± 12.99	374^b^.00 ± 12.86	390.50^ca^ ± 9.27	380.00^c^ ± 15.78	370.92^A^ ± 5.88
Group II	345.80^a^ ± 11.92	348.40^a^ ± 16.41	373.60^b^ ± 10.41	379.90^ba^ ± 10.93	378.00^b^ ± 15.94	365.14^A^ ± 6.11
PM	349.80^a^ ± 8.54	352.35^a^ ± 10.23	373.80^b^ ± 8.05	385.20^ba^ ± 7.08	379.00^b^ ± 10.92	
Glucose						
Group I	59.40^a^ ± 0.43	58.30^b^ ± 0.37	57.00^c^ ± 0.39	55.00^d^ ± 0.37	53.20^e^ ± 0.33	56.58^A^ ± 0.36
Group II	58.00^a^ ± 0.26	56.50^b^ ± 0.31	55.80^c^ ± 0.25	54.60^d^ ± 0.22	53.50^e^ ± 0.17	55.68^B^ ± 0.24
PM	58.70^a^ ± 0.29	57.40^b^ ± 0.31	56.40^c^ ± 0.27	54.80^d^ ± 0.21	53.35^e^ ± 0.18	

^A, B & a,b,c,d,e^ Means with different superscripts between columns and rows differ significantly (*p* < 0.05).

The difference in mean ± SE values of hematological parameters at different time points (−30, −15, 0, +15, +30 days of calving) in the transition feed trial group (Group I) and the control group (Group II) are shown in Table 5. The mean ± SE values of WBCs, lymphocytes, granulocytes, and MCV (mean corpuscular volume) depicted significant increases (*p* < 0.05) from −15 to +15 days, −15 to 0 day, −30 to +30 days, and −30 to 0 day, respectively. No significant differences (*p* < 0.05) were observed in the mean values of RBCs and MCH during the study period. However, significant decreases (*p* < 0.05) were observed in mean ± SE values of Hb (g/dl), HCT (hematocrit), and MCH from −30 to +15 days, −15 to 0 day, and+15 to +30 days, respectively (Table 5).

For WBC count, the LMM revealed significant main effects of Group (F(1, 18) = 7.89, *p* = 0.012), Time (F(4, 72) = 21.45, *p* < 0.001), and a significant Group × Time interaction (F(4, 72) = 3.56, *p* = 0.011). *Post hoc* Bonferroni-adjusted comparisons showed that Group I had significantly higher WBC counts than Group II at −30 days (mean difference = 0.07, *p* = 0.012), 0 day (mean difference = 0.25, *p* = 0.018), and +15 days (mean difference = 0.57, *p* < 0.001). No significant differences were observed at −15 days (*p* = 0.164) or +30 days (*p* = 0.526). Within both groups, WBC counts increased significantly from −30 to +30 days (Group I: +1.24, *p* < 0.001; Group II: +1.21, *p* < 0.001).

### 3.6 Effect of supplemented feed on biochemical parameters

Biochemical parameters, such as the serum level of albumin, total protein, and A:G ratio (0.77 ± 0.01 and 0.74 ± 0.01), increased significantly (*p* < 0.05) in Group I compared with Group II (Table 6). A similar trend was observed for blood serum calcium, cholesterol, and Ca: P ratio, which showed significant increases (*p* < 0.05) in Group I compared with Group II. Liver enzymes (SGOT; serum glutamic oxaloacetate transaminase and gamma-glutamyl transaminase), and BUN showed significant decreases (*p* < 0.05) in Group I compared with Group II (Table 8). The serum sodium level in Group I was significantly (*p* < 0.05) higher (137.67 ± 0.33) than in Group II (136.57 ± 0.37).

**Table 6:** Mean ± SE values of biochemical parameters in the transition feed and control groups at different time points during the study period.

Groups	Period in days					GM
	−30	−15	0	+15	+30	
Albumin (g/dl)						
Group I	3.15^a^ ± 0.07	3.27^b^ ± 0.06	3.07^c^ ± 0.06	2.90^d^ ± 0.04	2.93^d^ ± 0.08	3.06^A^ ± 0.03
Group II	3.16^a^ ± 0.07	3.09^b^ ± 0.06	2.96^c^ ± 0.07	2.79^d^ ± 0.04	2.84^d^ ± 0.05	2.97^B^ ± 0.03
PM	3.16^a^ ± 0.05	3.18^a^ ± 0.05	3.02^b^ ± 0.05	2.85^c^ ± 0.03	2.88^**c**^ ± 0.05	
Total protein (g/dl)						
Group I	7.13^a^ ± 0.05	7.20^b^ ± 0.05	7.04^c^ ± 0.05	6.91^d^ ± 0.04	7.07^e^ ± 0.04	7.07^A^ ± 0.02
Group II	7.14^a^ ± 0.07	7.11^a^ ± 0.07	6.94^b^ ± 0.06	6.84^c^ ± 0.05	6.87^c^ ± 0.05	6.98^B^ ± 0.03
PM	7.13^a^ ± 0.04	7.15^a^ ± 0.04	6.99^b^ ± 0.04	6.88^c^ ± 0.03	6.97^d^ ± 0.04	
Globulin (g/dl)						
Group I	3.98^a^ ± 0.06	3.94^b^ ± 0.05	3.97^c^ ± 0.05	4.01^d^ ± 0.05	4.14^e^ ± 0.08	4.01^A^ ± 0.03
Group II	3.97^a^ ± 0.03	4.02^b^ ± 0.03	3.98^c^ ± 0.04	4.05^d^ ± 0.03	4.03^d^ ± 0.02	4.09^**A**^ ± 0.01
PM	3.98^a^ ± 0.03	3.98^a^ ± 0.03	3.97^a^ ± 0.03	4.03^b^ ± 0.03	4.08^ca^ ± 0.04	
A:G ratio						
Group I	0.80 ± 0.03	0.83 ± 0.03	0.78 ± 0.02	0.73 ± 0.02	0.71 ± 0.03	0.77^A^ ± 0.01
Group II	0.80 ± 0.02	0.77 ± 0.02	0.75 ± 0.02	0.69 ± 0.01	0.71 ± 0.02	0.74^B^ ± 0.01
PM	0.80^a^ ± 0.02	0.80^a^ ± 0.02	0.76^b^ ± 0.02	0.71^c^ ± 0.01	0.71^c^ ± 0.02	
SGOT (U/L)						
Group I	98.13 ± 0.79	96.33 ± 0.99	99.35 ± 0.88	104.13 ± 1.24	110.76 ± 1.34	101.74^A^ ± 0.8
Group II	98.54 ± 0.92	100.88 ± 1.55	101.29 ± 0.30	110.86 ± 1.71	110.46 ± 1.40	104.41^B^ ± 0.9
PM	98.34^a^ ± 0.5	98.61^a^ ± 1.04	100.32^a^ ± 0.5	107.50^b^ ± 1.2	110.61^c^ ± 0.9	
SGPT (U/L)						
Group I	24.43 ± 0.55	23.76 ± 0.34	23.03 ± 0.22	24.97 ± 0.59	26.47 ± 0.48	24.53^A^ ± 0.26
Group II	25.81 ± 0.60	24.35 ± 0.39	23.88 ± 0.66	25.53 ± 0.56	26.06 ± 0.56	25.03^A^ ± 0.27
PM	25.12^a^ ± 0.43	24.06^b^ ± 0.26	23.45^c^ ± 0.35	25.25^d^ ± 0.40	26.27^e^ ± 0.36	
GGT (U/L)						
Group I	14.72 ± 0.37	14.32 ± 0.33	23.97 ± 0.33	20.97 ± 0.69	20.75 ± 0.42	18.94^A^ ± 0.57
Group II	16.04 ± 0.37	16.59 ± 0.36	24.17 ± 0.29	21.87 ± 0.46	21.64 ± 0.45	20.06^B^ ± 0.49
PM	15.38^a^ ± 0.30	15.45^a^ ± 0.35	24.07^b^ ± 0.21	21.42^c^ ± 0.41	21.19^ca^ ± 0.32	
Cholesterol (mg/dl)						
Group I	79.03 ± 1.56	78.72 ± 0.42	71.64 ± 1.15	78.73 ± 1.14	80.40 ± 1.14	77.71^A^ ± 0.64
Group II	76.94a ± 0.71	74.89^b^ ± 0.37	68.82 ± 1.09	73.45 ± 0.29	78.76 ± 0.86	74.57^B^ ± 0.58
						
PM	77.98^a^ ± 0.87	76.81^a^ ± 0.52	70.23^b^ ± 0.83	76.09^c^ ± 0.83	79.58^d^ ± 0.59	
BUN (mg/dl)						
Group I	10.96 ± 0.25	10.89 ± 0.35	11.02 ± 0.27	12.06 ± 0.41	11.11 ± 0.27	11.21^A^ ± 0.15
Group II	11.85 ± 0.54	10.85 ± 0.28	11.79 ± 0.49	11.66 ± 0.30	12.97 ± 0.56	11.82^B^ ± 0.22
PM	11.41^a^ ± 0.31	10.87^b^ ± 0.22	11.40^c^ ± 0.29	11.86^d^ ± 0.25	12.04^d^ ± 0.37	
Creatinine (mg/dl)						
Group I	1.20 ± 0.01	1.18 ± 0.01	1.24 ± 0.01	1.23 ± 0.01	1.24 ± 0.01	1.21^A^ ± 0.01
Group II	1.21 ± 0.02	1.21 ± 0.03	1.24 ± 0.02	1.23 ± 0.02	1.22 ± 0.02	1.22^A^ ± 0.01
PM	1.20^a^ ± 0.01	1.20^a^ ± 0.01	1.24^b^ ± 0.01	1.23^b^ ± 0.01	1.23^b^ ± 0.01	
Triglyceride (mg/dl)						
Group I	25.30 ± 0.52	25.47 ± 0.55	22.48 ± 0.85	15.17 ± 0.24	14.22 ± 0.22	20.53^A^ ± 0.73
Group II	25.25 ± 0.65	23.13 ± 0.58	15.37 ± 0.37	15.13 ± 0.33	13.82 ± 0.23	19.54^A^ ± 0.70
PM	25.27 ± 0.41^a^	24.30^a^ ± 0.47	18.93^b^ ± 0.93	15.15^c^ ± 0.20	14.02^c^ ± 0.16	
HDL (mg/dl)						
Group I	41.05^a^ ± 0.79	39.74^b^ ± 0.75	38.68^c^ ± 0.67	43.82 ± 0.40	45.77 ± 0.36	41.81^A^ ± 0.46
Group II	41.49 ± 0.69	38.86 ± 0.44	40.35 ± 0.69	45.04 ± 0.48	44.41 ± 0.29	42.03^A^ ± 0.41
PM	41.27^a^ ± 0.51	39.30^b^ ± 0.44	39.51^b^ ± 0.51	44.43^c^ ± 0.33	45.09^c^ ± 0.27	
Calcium (mg/dl)						
Group I	8.29 ± 0.09	8.52 ± 0.09	8.23 ± 0.04	8.06 ± 0.03	7.94 ± 0.05	8.20^A^ ± 0.04
Group II	8.20 ± 0.10	8.24 ± 0.08	8.08 ± 0.05	7.99 ± 0.05	7.89 ± 0.07	8.08^B^ ± 0.04
PM	8.24^a^ ± 0.06	8.38^b^ ± 0.07	8.15^c^ ± 0.04	8.02^d^ ± 0.03	7.91^e^ ± 0.04	
Sodium (mEq/l)						
Group I	137.85 ± 0.77	139.12 ± 0.76	138.97 ± 0.44	136.57 ± 0.55	135.84 ± 0.56	137.67^A^ ± 0.33
Group II	137.82 ± 0.84	136.57 ± 0.99	138.21 ± 0.76	135.43 ± 0.50	134.82 ± 0.51	136.57^B^ ± 0.37
PM	137.83^a^ ± 0.55	137.84^a^ ± 0.67	138.59^b^ ± 0.44	136.00^c^ ± 0.38	135.33^d^ ± 0.39	
Potassium (mEq/l)						
Group I	3.93 ± 0.05	4.02 ± 0.05	3.96 ± 0.06	3.71 ± 0.04	3.65 ± 0.03	3.85^A^ ± 0.03
Group II	3.77 ± 0.05	3.77 ± 0.04	3.85 ± 0.06	3.69 ± 0.03	3.63 ± 0.03	3.74^A^ ± 0.02
PM	3.85^a^ ± 0.04	3.89^a^ ± 0.04	3.91^a^ ± 0.04	3.70^a^ ± 0.03	3.64^ba^ ± 0.02	
Magnesium (mEq/l)						
Group I	1.20 ± 0.03	1.22 ± 0.03	1.17 ± 0.01	1.18 ± 0.02	1.15 ± 0.02	1.18^A^ ± 0.01
Group II	1.18 ± 0.01	1.19 ± 0.02	1.17 ± 0.02	1.14 ± 0.02	1.18 ± 0.01	1.17^A^ ± 0.01
PM	1.19^a^ ± 0.02	1.20^a^ ± 0.02	1.17^b^ ± 0.01	1.16^b^ ± 0.01	1.17^ba^ ± 0.01	
Phosphorus (mg/dl)						
Group I	4.66 ± 0.09	4.70 ± 0.08	4.73 ± 0.05	4.64 ± 0.04	4.63 ± 0.07	4.67^A^ ± 0.03
Group II	4.90 ± 0.03	4.85 ± 0.03	4.93 ± 0.03	4.69 ± 0.04	4.78 ± 0.03	4.83^B^ ± 0.02
PM	4.78^a^ ± 0.06	4.77^a^ ± 0.04	4.83^b^ ± 0.03	4.66^c^ ± 0.03	4.71^a^ ± 0.04	
Ca:P						
Group I	1.79 ± 0.04	1.82 ± 0.04	1.74 ± 0.02	1.74 ± 0.02	1.72 ± 0.03	1.76^A^ ± 0.01
Group II	1.67 ± 0.02	1.70 ± 0.02	1.64 ± 0.01	1.71 ± 0.01	1.65 ± 0.02	1.67^B^ ± 0.01
PM	1.73^a^ ± 0.03	1.76^b^ ± 0.03	1.69^c^ ± 0.02	1.72^d^ ± 0.01	1.68^e^ ± 0.02	

^A, B&a,b,c,d,e^ Means with different superscripts between columns and rows differ significantly (*p* < 0.05).

**Table 8:** Difference in fold expression of the NUAK1 gene between the transition feed and control groups at different time points in buffaloes.

Groups	Transition period in days				GM
	−15	0	+15	+30	
Group I (supplemented)	0.908^a^ ± 0.03	0.937^a^ ± 0.05	1.027^b^ ± 0.04	1.116^c^ ± 0.02	0.952^a^ ± 0.04
Group II (control)	0.918^a^ ± 0.04	1.083^b^ ± 0.02	1.032^c^ ± 0.01	1.179^d^ ± 0.15	1.297^b^ ± 0.02

^A, B&a,b,c,d,e^ Means with different superscripts between columns and rows differ significantly (*p* < 0.05).

The LMM revealed significant Group effects for albumin (F(1, 18) = 5.67, *p* = 0.028), total protein (F(1, 18) = 6.89, *p* = 0.017), calcium (F(1, 18) = 8.12, *p* = 0.010), and phosphorus (F(1, 18) = 14.56, *p* = 0.001). Significant Group × Time interactions were observed for albumin (F(4, 72) = 3.21, *p* = 0.017), total protein (F(4, 72) = 2.98, *p* = 0.024), and calcium (F(4, 72) = 3.45, *p* = 0.012).

### 3.7 Effect of transition feed on BHBA (β-hydroxybutyric assay) and cytokine (TNF-α and interferon-γ) levels using ELISA

The mean ± SE values of BHBA, TNF-α, and IFN-γ decreased significantly (*p* < 0.05) in the transition feed trial group (Group I) compared with the control group (Group II) (Table 9). The main effects of Group (F(1, 18) = 26.78, *p* < 0.001) and Time (F(4, 72) = 14.92, *p* < 0.001), along with a significant Group × Time interaction (F(4, 72) = 6.31, *p* < 0.001), were observed. *Post hoc* Bonferroni-adjusted comparisons showed that BHBA concentrations were significantly lower in Group I than in Group II at all time points: −30 days (mean difference = −13.48, *p* = 0.011), −15 days (mean difference = −44.63, *p* < 0.001), 0 day (mean difference = −71.12, *p* < 0.001), +15 days (mean difference = −93.61, *p* < 0.001), and +30 days (mean difference = −110.37, *p* < 0.001). Within both groups, BHBA increased significantly from −30 to +30 days (Group I: +27.55, *p* < 0.001; Group II: +124.44, *p* < 0.001). However, the magnitude of increase was substantially lower in the supplemented group, indicating that transition feed supplementation effectively attenuated the postpartum rise in BHBA associated with NEB.

**Table 9:** Mean ± SE values of BHBA, TNF-α, and INF-γ in the transition feed and control groups at different time points during the study period.

Groups	Period in days					GM
	−30	−15	0	+15	+30	
TNF-α (ng/ml)						
Group I	1.10^a^ ± 0.03	1.19^a^ ± 0.07	2.07^b^ ± 0.03	1.84^c^ ± 0.06	1.74^c^ ± 0.04	1.59^A^ ± 0.06
Group II	1.54^a^ ± 0.02	2.05^b^ ± 0.07	2.27^c^ ± 0.07	2.25^c^ ± 0.12	2.07^d^ ± 0.12	2.03^B^ ± 0.05
PM	1.32^a^ ± 0.05	1.62^b^ ± 0.11	2.17^c^ ± 0.04	2.05^c^ ± 0.08	1.90^c^ ± 0.07	
BHBA (nmol/ml)						
Group I	298.36^a^ ± 4.2	293^a^.17 ± 3.4	300^a^.90 ± 3.49	315^ab^.94 ± 3.58	325^b^.91 ± 3.20	306.86^A^ ± 2.3
Group II	311.84^a^ ± 5.29	337.80^b^ ± 9.76	372.02^c^ ± 17.34	409.55^d^ ± 32.93	436.28^e^ ± 27.87	373.50^B^ ± 11.2
PM	305.10^a^ ± 3.65	315.48^a^ ± 7.18	336.46^b^ ± 11.86	362.74^c^ ± 19.37	381.10^ca^ ± 18.62	
INF-γ (ng/ml)						
Group I	0.71^a^ ± 0.03	0.74^a^ ± 0.01	1.08^b^ ± 0.03	1.12^bc^ ± 0.05	1.06^b^ ± 0.03	0.94^A^ ± 0.03
Group II	0.83^a^ ± 0.02	0.92^a^ ± 0.06	1.14^b^ ± 0.04	1.14^b^ ± 0.05	1.09^ba^ ± 0.05	1.02^B^ ± 0.03
PM	0.77^a^ ± 0.02	0.83^b^ ± 0.04	1.11^c^ ± 0.03	1.13^c^ ± 0.03	1.07^d^ ± 0.03	

^A,B & a,b,c,d,e^ Means with different superscripts between columns and rows differ significantly (*p* < 0.05).

For TNF-α, the LMM revealed significant main effects of Group (F(1, 18) = 34.56, *p* < 0.001) and Time (F(4, 72) = 42.89, *p* < 0.001), along with a significant Group × Time interaction (F(4, 72) = 5.67, *p* < 0.001). *Post hoc* Bonferroni-adjusted comparisons showed that TNF-α concentrations were significantly lower in Group I than in Group II at all time points: −30 days (mean difference = −0.44, *p* < 0.001), −15 days (mean difference = −0.86, *p* < 0.001), 0 day (mean difference = −0.20, *p* = 0.002), +15 days (mean difference = −0.41, *p* < 0.001), and +30 days (mean difference = −0.33, *p* = 0.002). Within both groups, TNF-α increased significantly from −30 to 0 day (Group I: +0.97, *p* < 0.001; Group II: +0.73, *p* < 0.001) and then declined. The consistently lower TNF-α levels in the supplemented group indicate reduced systemic inflammation during the transition period.

For IFN-γ, the LMM revealed significant main effects of Group (F(1, 18) = 6.78, *p* = 0.018) and Time (F(4, 72) = 34.56, *p* < 0.001), along with a significant Group × Time interaction (F(4, 72) = 3.12, *p* = 0.020). *Post hoc* Bonferroni-adjusted comparisons showed that IFN-γ concentrations were significantly lower in Group I than in Group II at −30 days (mean difference = −0.12, *p* < 0.001) and −15 days (mean difference = −0.18, *p* < 0.001). No significant differences were observed at 0 day (*p* = 0.104), +15 days (*p* = 0.695), or +30 days (*p* = 0.475). Within both groups, IFN-γ increased significantly from −30 to 0 day (Group I: +0.37, *p* < 0.001; Group II: +0.31, *p* < 0.001) and remained elevated through +30 days. The lower IFN-γ levels in the supplemented group during the prepartum period suggest reduced inflammatory activation.

### 3.8 Effect of supplemented feed on oxidative stress parameters

The transition feed group (Group I) showed a significant decrease (*p* < 0.05) in lipid peroxidation (LPO) compared with the control group (Group II). In contrast, the levels of antioxidant markers such as glutathione peroxidase (GPx), superoxide dismutase (SOD), and catalase (CAT) were significantly higher (*p* < 0.05) in Group I, as depicted in Table 7.

The mean ± SE values of LPO at different time points during the study, i.e., −30, −15, 0, 15, and 30 days, showed significant differences (*p* < 0.05) over the period from −30 to +30 days, with the highest value at 0 day; thereafter, a reduction in concentration was observed (Table 7).

**Table 7:** Mean ± SE values of oxidative biomarkers in the transition feed and control groups at different time points during the study period.

Groups	Transition period in days					GM
	−30	−15	0	+15	+30	
LPO						
Group I	5.37^a^ ± 0.21	5.09^b^ ± 0.07	5.66^c^ ± 0.07	5.31^d^ ± 0.05	5.12^d^ ± 0.04	5.31^a^ ± 0.04
Group II	5.71^a^ ± 0.04	5.41^b^ ± 0.04	5.82^c^ ± 0.02	5.59^d^ ± 0.04	5.42^e^ ± 0.04	5.59^b^ ± 0.04
PM	5.54^a^ ± 0.11	5.25^b^ ± 0.05	5.74^c^ ± 0.04	5.45^d^ ± 0.05	5.27^e^ ± 0.04	
GPX						
Group I	3.23^a^ ± 0.06	3.16^a^ ± 0.06	2.28^b^ ± 0.04	2.00^c^ ± 0.05	2.30^d^ ± 0.04	2.59^a^ ± 0.07
Group II	2.99^a^ ± 0.04	2.88^a^ ± 0.03	1.96^b^ ± 0.12	1.84^c^ ± 0.04	2.04^d^ ± 0.03	2.34^b^ ± 0.07
PM	3.11^**a**^ ± 0.04	3.02^**a**^ ± 0.05	2.12^c^ ± 0.07	1.92^d^ ± 0.04	2.17^b^ ± 0.04	
SOD						
Group I	43.42^a^ ± 0.84	41.69^b^ ± 0.60	40.95^b^ ± 0.40	39.28^c^ ± 0.45	38.30^c^ ± 0.72	40.73^a^ ± 0.37
Group II	42.53^a^ ± 0.79	39.40^b^ ± 0.62	37.22^c^ ± 0.50	35.92^d^ ± 0.44	34.51^e^ ± 0.69	37.91^b^ ± 0.48
PM	42.97^a^ ± 0.57	40.55^b^ ± 0.50	39.08^c^ ± 0.53	37.60^d^ ± 0.49	36.40^e^ ± 0.65	
Catalase						
Group I	125.61^a^ ± 0.37	126.66^a^ ± 0.37	124.59^b^ ± 0.67	121.10^c^ ± 0.79	119.12^d^ ± 0.54	123.41^a^ ± 0.47
Group II	123.97^a^ ± 0.47	125.07^a^ ± 0.34	123.18^b^ ± 0.52	119.29^c^ ± 0.56	117.84^d^ ± 0.72	121.87^b^ ± 0.46
PM	124.79^a^ ± 0.34	125.86^a^ ± 0.31	123.88^b^ ± 0.44	120.20^c^ ± 0.51	118.48^d^ ± 0.46	

For LPO, the LMM revealed significant main effects of Group (F(1, 18) = 12.34, *p* = 0.002), Time (F(4, 72) = 8.67, *p* < 0.001), and the Group × Time interaction (F(4, 72) = 2.89, *p* = 0.028). For SOD, significant Group (F(1, 18) = 15.78, *p* < 0.001), Time (F(4, 72) = 11.23, *p* < 0.001), and Group × Time interaction (F(4, 72) = 3.56, *p* = 0.010) effects were observed.

For GPX, the LMM revealed significant main effects of Group (F(1, 18) = 12.34, *p* = 0.002) and Time (F(4, 72) = 56.78, *p* < 0.001), along with a significant Group × Time interaction (F(4, 72) = 3.89, *p* = 0.006). *Post hoc* Bonferroni-adjusted comparisons showed that GPX activity was significantly higher in Group I than in Group II at all time points: −30 days (mean difference = +0.24, *p* < 0.001), −15 days (mean difference = +0.28, *p* < 0.001), 0 day (mean difference = +0.32, *p* = 0.002), +15 days (mean difference = +0.16, *p* = 0.002), and +30 days (mean difference = +0.26, *p* < 0.001). Within both groups, GPX activity decreased significantly from −30 to +15 days (Group I: −1.23, *p* < 0.001; Group II: −1.15, *p* < 0.001) and then partially recovered by +30 days. The higher GPX activity in the supplemented group indicates enhanced antioxidant capacity.

For catalase, the LMM revealed significant main effects of Group (F(1, 18) = 8.45, *p* = 0.009) and Time (F(4, 72) = 45.67, *p* < 0.001), along with a significant Group × Time interaction (F(4, 72) = 2.56, *p* = 0.045). *Post hoc* Bonferroni-adjusted comparisons showed that catalase activity was significantly higher in Group I than in Group II at −30 days (mean difference = +1.64, *p* = 0.001), −15 days (mean difference = +1.59, *p* < 0.001), 0 day (mean difference = +1.41, *p* = 0.030), and +15 days (mean difference = +1.81, *p* = 0.017). At +30 days, a tendency toward higher catalase activity was observed in Group I (mean difference = +1.28, *p* = 0.060). Within both groups, catalase activity decreased significantly from −30 to +30 days (Group I: −6.49, *p* < 0.001; Group II: −6.13, *p* < 0.001). The higher catalase activity in the supplemented group indicates enhanced antioxidant defense against oxidative stress during the transition period.

### 3.9 Effect of transition feed on the expression levels of hypocalcemia/milk fever-specific genes

**Figure 2:**
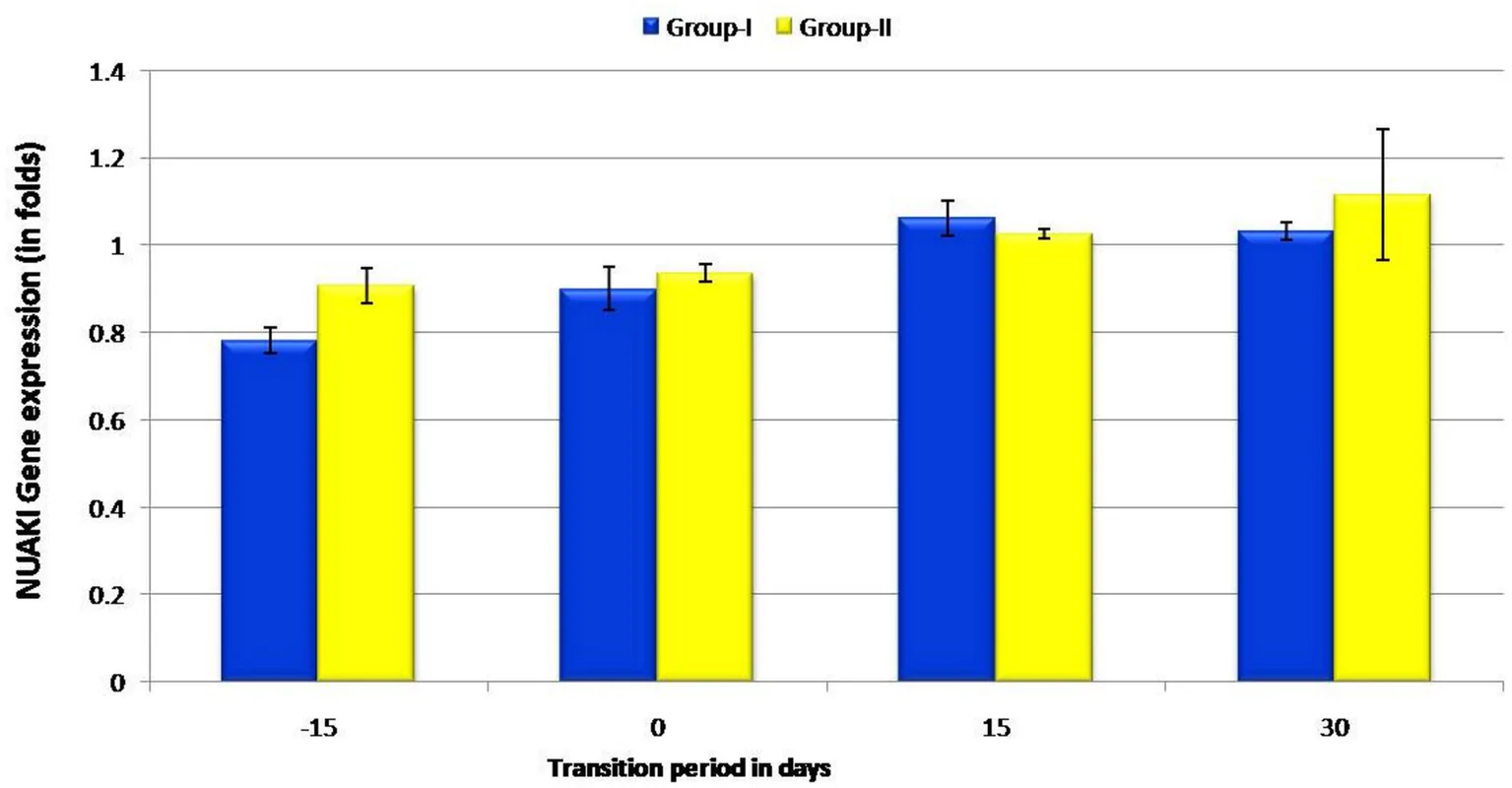
Changes in the expression level of the NUAK1 gene between the transition feed and control groups at different time points during the transition period in buffaloes.

In this study, a significant decrease (*p* < 0.05) in the expression level of the NUAK1 gene was observed in the transition feed group compared with the control feed group. Across the different time points during the transition period, NUAK1 expression increased significantly (*p* < 0.05) from −15 to +30 days in both groups (Figure 2). The fold-change values for gene expression are presented in Table 8. LMM revealed a significant *Group × Time interaction* (F(3, 54) = 3.08, *p* = 0.035), indicating that the pattern of NUAK1 expression over time differed between the supplemented and control groups. *Post hoc* Bonferroni-adjusted comparisons showed that NUAK1 expression was significantly lower in Group I than in Group II at 0 day (fold change: 0.937 vs. 1.083; mean difference = −0.146, *p* = 0.001). No significant differences were observed at −15 days (*p* = 0.779), +15 days (*p* = 0.865), or +30 days (*p* = 0.564). Within both groups, NUAK1 expression increased significantly from −15 to +30 days (Group I: +0.208, *p* < 0.001; Group II: +0.261, *p* = 0.040). The lower expression of NUAK1 in the supplemented group at calving (day 0) suggests a reduced risk of hypocalcemia/milk fever.

**Figure 3:**
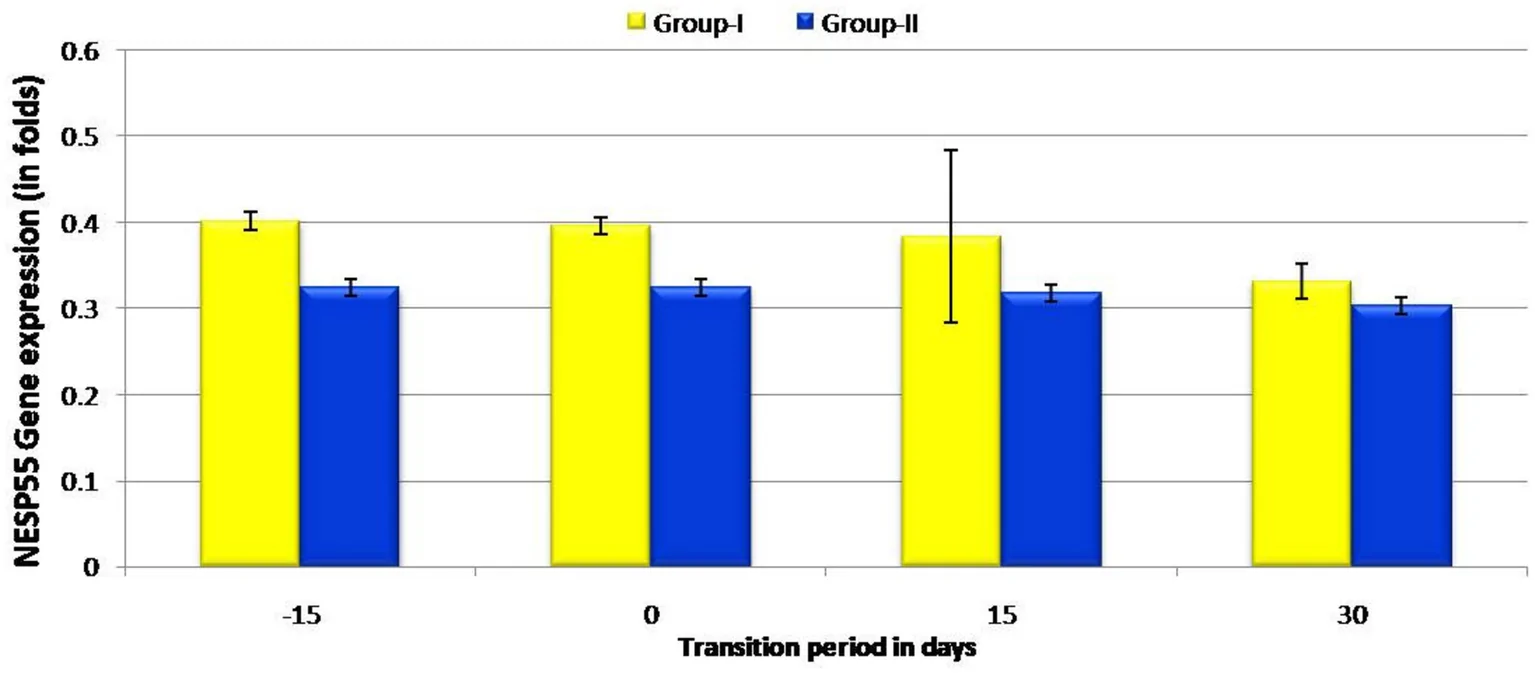
Changes in the expression level of the NESP55 gene between the transition feed and control groups at different time points during the transition period in buffaloes.

NESP55 (neuroendocrine secretory protein 55) is a downregulated gene associated with the risk of hypocalcemia/milk fever. In this study, the expression level of NESP55 was significantly higher (*p* < 0.05) in the transition feed-supplemented group (Group I) than in the control group (Group II) at −15 days (fold change: 0.402 vs. 0.348; mean difference = +0.054) and at 0 day (fold change: 0.396 vs. 0.334; mean difference = +0.062). At +15 days, although Group I maintained higher expression (0.384 vs. 0.318), the difference was not statistically significant (*p* = 0.364). At +30 days, a tendency toward higher expression was observed in Group I (0.332 vs. 0.303; *p* = 0.087) (Figure 3).

For NESP55 gene expression, the LMM revealed significant main effects of Group (F(1, 18) = 8.45, *p* = 0.009), Time (F(3, 54) = 6.78, *p* < 0.001), and a significant Group × Time interaction (F(3, 54) = 2.89, *p* = 0.044). These findings indicate that NESP55 expression was consistently higher in the supplemented group and that the pattern of change over the transition period differed between the two groups.

*Post hoc* Bonferroni-adjusted comparisons showed that NESP55 expression was significantly higher in Group I than in Group II at −15 days (mean difference = +0.054, 95% CI: 0.034 to 0.074, *p* < 0.001) and at 0 day (mean difference = +0.062, 95% CI: 0.042 to 0.082, *p* < 0.001). No significant difference was observed at +15 days (mean difference = +0.066, 95% CI: −0.076 to 0.208, *p* = 0.364), while a tendency was noted at +30 days (mean difference = +0.029, 95% CI: −0.003 to 0.061, *p* = 0.087) (see Table 11).

**Table 11:** Difference in the fold of expression of the NESP55 gene between the transition feed and control groups.

Groups	Transition period in days				GM
	−15	0	15	30	
Group I (supplemented)	0.402^c^ ± 0.01	0.396^c^ ± 0.01	0.384^b^ ± 0.1	0.332^a^ ± 0.02	0.379^a^ ± 0.01
Group II (control)	0.348^d^ ± 0.01	0.334^c^ ± 0.01	0.318^b^ ± 0.01	0.303^a^ ± 0.01	0.318^b^ ± 0.01

^A,B & a,b,c,d,e^ Means with different superscripts between columns and rows differ significantly (*p* < 0.05).

### 3.10 Effect of transition feed on the expression level of negative energy balance/ketosis-specific genes

**Figure 4:**
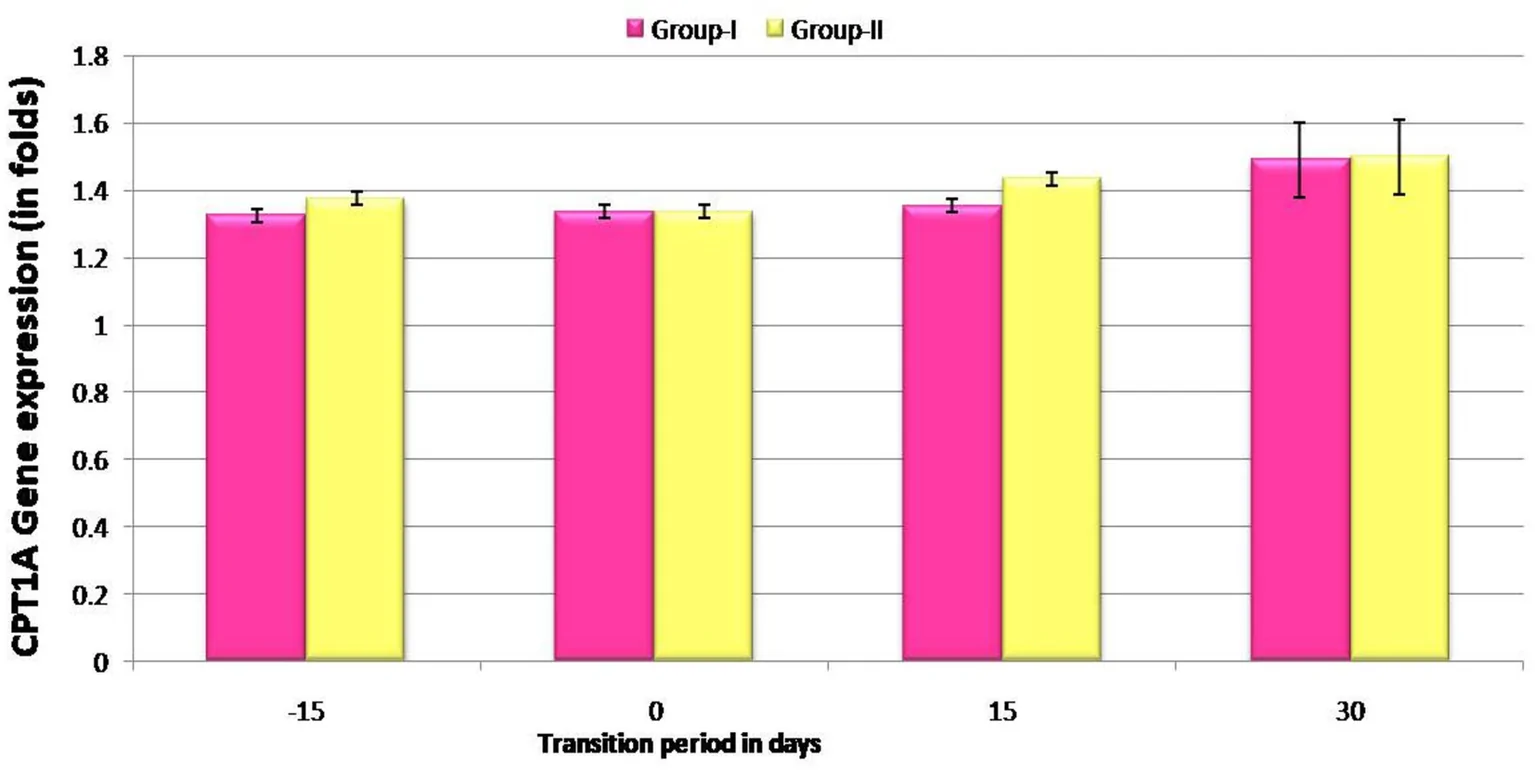
Changes in the expression level of the CPT1A gene between the transition feed and control groups at different time points during the transition period in buffaloes.

In this study, there was a significant decrease (*p* < 0.05) in the expression level of CPTIA (an upregulated gene) in the transition feed group compared with the control group (Table 10). These results suggest that supplementation with transition feed reduces the probability of NEB/ketotic conditions in buffaloes. There was a significant increase (*p* < 0.05) in the expression level of CPT1A as the transition period progressed from −30 to +30 days in both groups (Figure 4). LMM revealed significant main effects of Group (F(1, 18) = 4.56, *p* = 0.047), Time (F(3, 54) = 9.23, *p* < 0.001), and a significant Group × Time interaction (F(3, 54) = 2.89, *p* = 0.044).

**Table 10:** Difference in fold expression of the CPT1A gene between the transition feed and control groups at different time points in the buffaloes.

Groups	Transition period in days				GM
	−15	0	+15	+30	
Group I (supplemented)	1.323^a^ ± 0.02	1.335^b^ ± 0.02	1.353^c^ ± 0.02	1.489^d^ ± 0.11	1.123^A^ ± 0.05
Group II (control)	1.375^a^ ± 0.02	1.336^b^ ± 0.02	1.433^c^ ± 0.02	1.499^c^ ± 0.11	1.378^B^ ± 0.03

^A,B & a,b,c,d,e^ Means with different superscripts between columns and rows differ significantly (*p* < 0.05).

**Figure 5:**
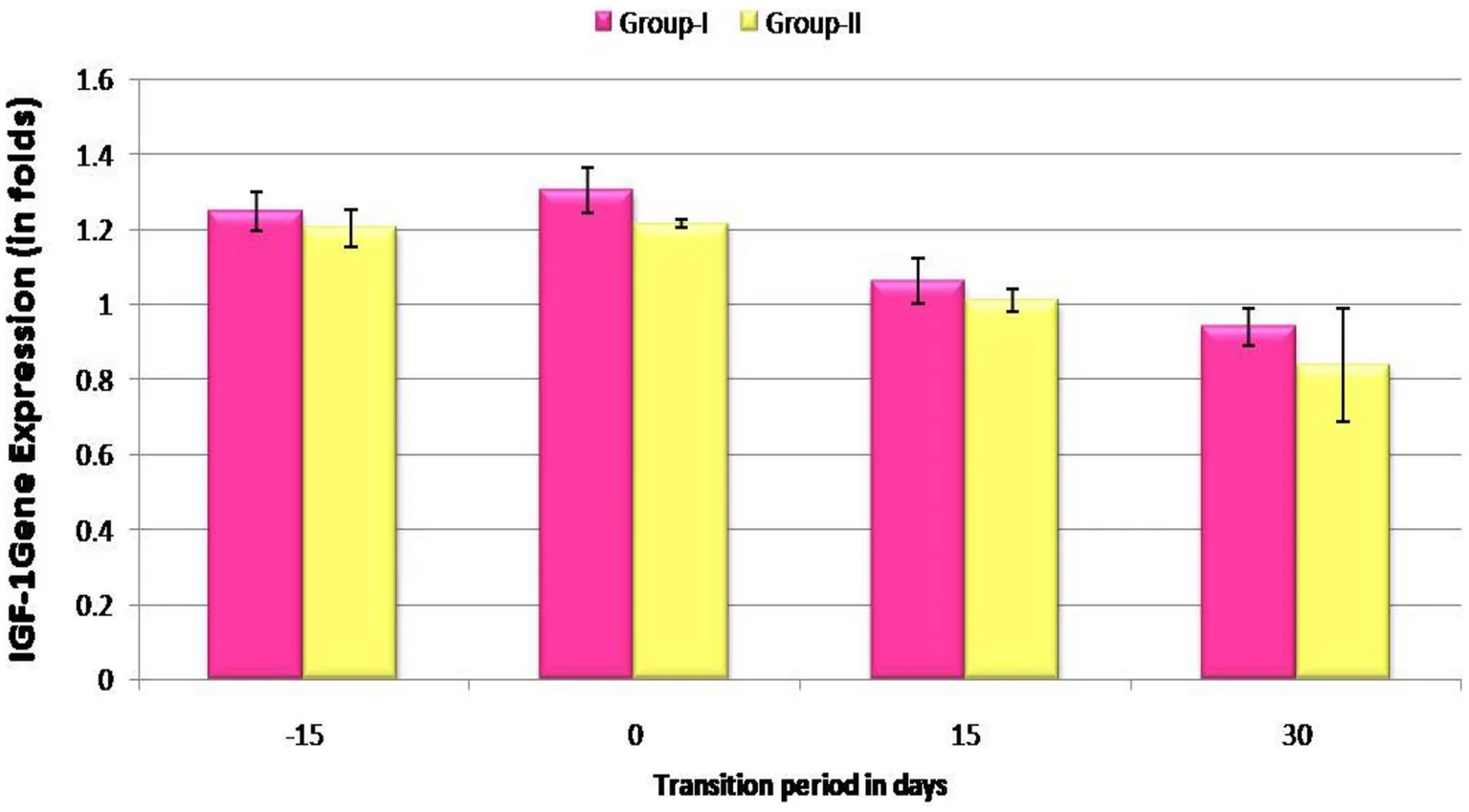
Changes in the expression level of the IGF-1 gene between the transition feed and control groups at different time points during the transition period in buffaloes.

In this study, a significant increase (*p* < 0.05) in the expression level of IGF-1 (a downregulated gene) was observed in the transition feed group compared with the control group, which suggests that increased expression of IGF-1 in the supplemented group may reduce the probability of negative energy balance/ketotic conditions in buffaloes (Table 12). Across the different time points, there was a significant decrease (*p* < 0.05) in the expression level of IGF-1 as the transition period progressed from −30 to +30 days (Figure 5). LMM revealed a significant main effect of Group (F(1, 18) = 5.12, *p* = 0.036), a significant main effect of Time (F(3, 54) = 12.45, *p* < 0.001), and a significant Group × Time interaction (F(3, 54) = 3.21, *p* = 0.030).

**Table 12:** Difference in fold expression of the IGF-1 gene between the transition feed and control groups at different time points in buffaloes.

Groups	Transition period in days				GM
	−15	0	15	30	
Group I (supplemented)	1.247^c^ ± 0.05	1.304^c^ ± 0.06	1.063^b^ ± 0.06	0.939^a^ ± 0.05	0.974^A^ ± 0.06
Group II (control)	1.203^d^ ± 0.05	1.214^c^ ± 0.01	1.008^b^ ± 0.03	0.838 ± 0.15	0.905^A^ ± 0.04

^A,B & a,b,c,d,e^ Means with different superscripts between columns and rows differ significantly (*p* < 0.05).

### 3.11 Effect of transition feed on the expression level of cytokines

**Figure 6:**
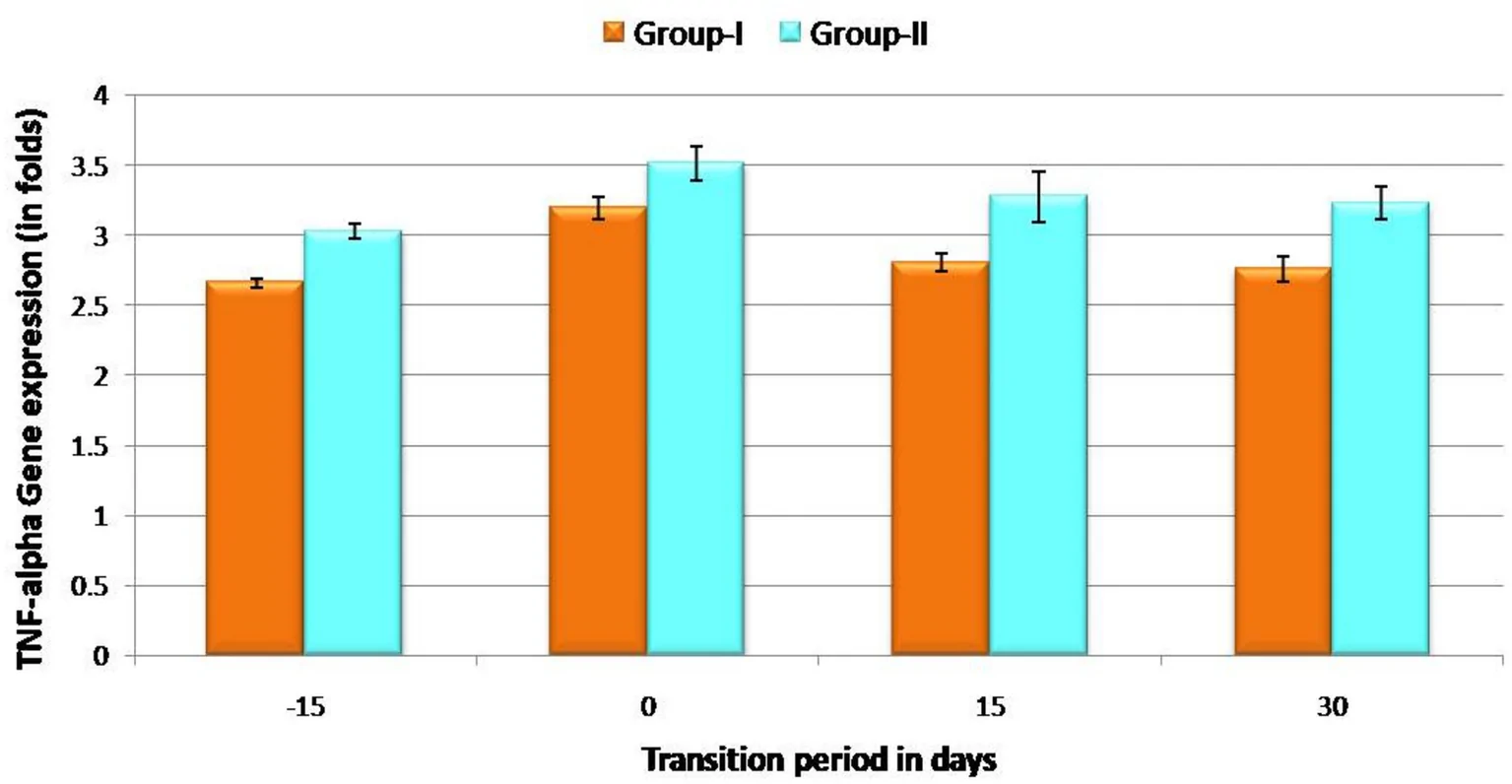
Changes in the expression level of the TNF-α gene between the transition feed and control groups at different time points during the transition period in buffaloes.

**Figure 7:**
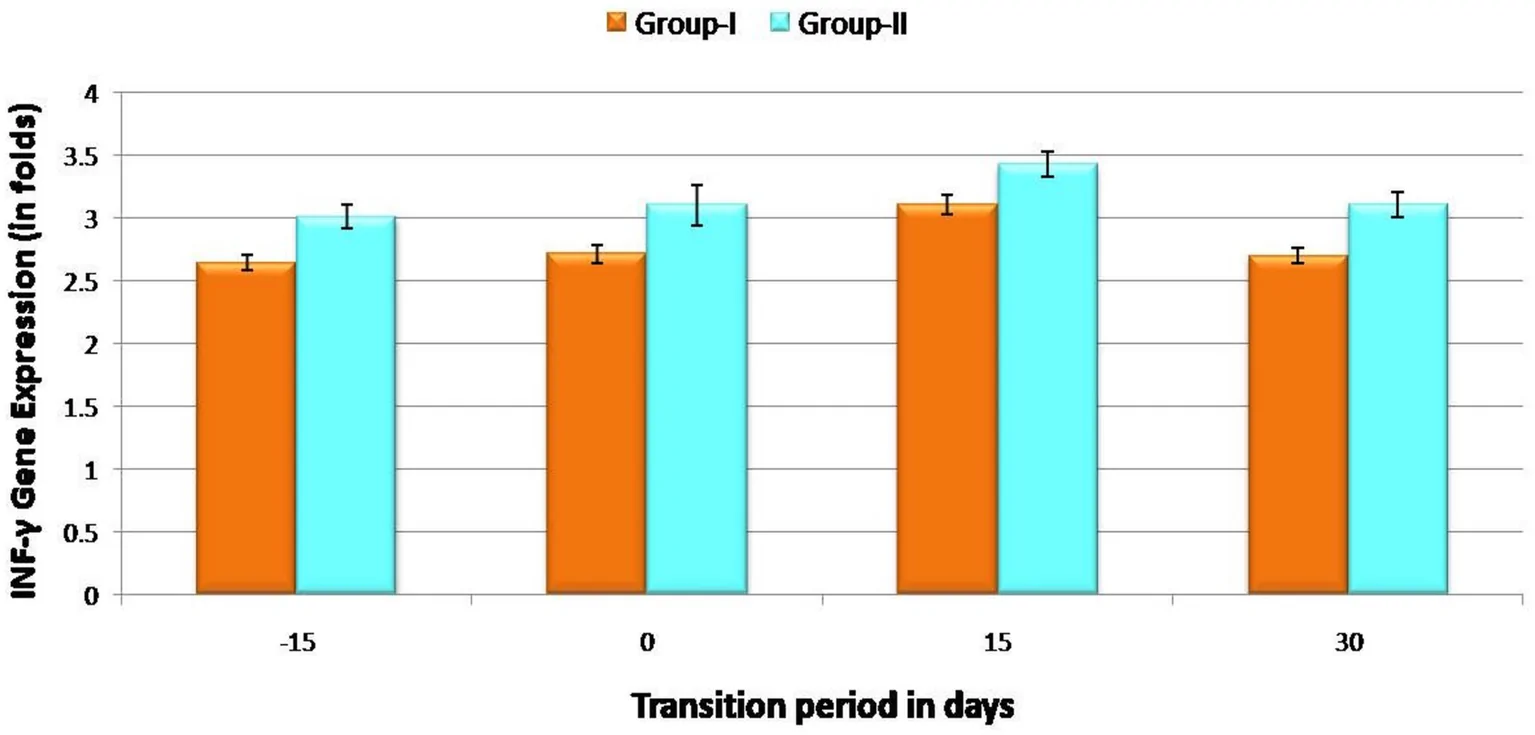
Changes in the expression level of the IFN-γ gene between the transition feed and control groups at different time points during the transition period in buffaloes.

It is well established that during the transition period, the inflammatory response is elicited as animals approach calving and during the postpartum period, which increases the expression of inflammatory markers, e.g., TNF-α and IFN-γ. This study revealed a significant decrease (*p* < 0.05) in the expression level of TNF-α (an upregulated gene associated with inflammation) in the feed group than in the control group (Table 13). There was also a significant increase (*p* < 0.05) in the expression level of TNF-α as the transition period progressed, i.e., from −15 to +30 days (Figure 6). A similar trend was observed in the expression level of INF-γ between the feed and control groups, as shown in Table 14 (Figure 7). LMM revealed significant main effects of Group (F(1, 18) = 7.89, *p* = 0.011), Time (F(3, 54) = 15.34, *p* < 0.001), and the Group × Time interaction (F(3, 54) = 3.67, *p* = 0.017).

**Table 13:** Difference in fold expression of the TNF-α gene between the transition feed and control groups at different time points in buffaloes.

Groups	Transition period in days				GM
	−15	0	15	30	
Group I (supplemented)	2.658^a^ ± 0.09	3.195^b^ ± 0.08	2.802^c^ ± 0.06	2.757^c^ ± 0.06	2.853 ± 0.06
Group II (control)	3.028^a^ ± 0.08	3.512^b^ ± 0.12	3.275^c^ ± 0.18	3.227^c^ ± 0.12	3.274 ± 0.07

^A,B & a,b,c,d,e^ Means with different superscripts between columns and rows differ significantly (*p* < 0.05).

**Table 14:** Difference in fold expression of the INF-γ gene between the transition feed and control groups at different time points in buffaloes.

Groups	−15	0	15	30	GM
Group I (supplemented)	2.645^a^ ± 0.06	2.715^a^ ± 0.07	3.108^b^ ± 0.08	2.702^c^ ± 0.06	2.840 ± 0.04
Group II (control)	3.015^a^ ± 0.09	3.107^a^ ± 0.16	3.43^b^ ± 0.10	3.113^c^ ± 0.10	3.120 ± 0.06

^A,B & a,b,c,d,e^ Means with different superscripts between columns and rows differ significantly (*p* < 0.05).

## 4 Discussion

Our study evaluated the effects of nutritional intervention during the transition period in buffaloes in terms of BCS, hematobiochemical and oxidative parameters, and molecular markers associated with hypocalcemia, NEB, and inflammatory pathways. The transition period is a critical period, encompassing various endocrinological, metabolic, and reproductive changes that make dairy animals prone to various production and infectious diseases. Consistent with previous studies highlighting the importance of the transition period in dairy cows, limited data are available on buffaloes, particularly with regard to nutritional requirements during this period.

Metabolic adaptation, increased nutrient demands, insulin resistance, and lipolytic changes during the postpartum period induce a state of NEB (7). The transition feed was strategically formulated to provide a balanced combination of energy, digestible fiber, high-quality protein, rumen-protected protein, minerals, vitamins, and buffering agents to meet the elevated metabolic demands in buffaloes during the transition period. The chemical composition of the transition feed included 17% crude fiber and 20.8% crude protein. Proteins, however, stimulate the growth of cellulolytic bacteria in the rumen and increase fiber digestibility and feed intake (20). A few studies documenting the use of similar ingredients (individually or in combination) have been reported in dairy cattle during the transition period. Iqbal et al. (74) reported that the use of 10% wheat bran with straw maintained dry matter intake and energy balance and improved milk production during the first 3 weeks of lactation. Turk et al. (75) conducted a study in Holstein-Friesian (HF) cattle to investigate the effect of a diet containing grass silage with dry matter (DM) content varying from 35 to 45%, crude protein content from 13 to 15%, crude fiber content from 25 to 30%, crushed maize grains, and a vitamin–mineral mixture on lipid mobilization. They found comparatively lower concentrations of free fatty acids and β-hydroxybutyric acid. The synergistic effects of vitamin E and selenium have been reported to protect against free radicals and to protect neutrophils against oxidative damage, thereby boosting the host immune system (76). Collectively, these findings provide a physiological and nutritional rationale for the strategic formulation of transition feed to maintain metabolic and immune stability during this period. The beneficial effects of improved nutritional status during the transition period can be reflected in changes in BCS and subsequent lactation performance. The BCS has been considered a valuable tool for managing nutritional strategies and assessing the occurrence of metabolic disorders (21). BCS loss is associated with declines in milk production, increases in circulating non-esterified fatty acids (NEFA) levels, and reproductive disorders. A greater degree of BCS loss during the postpartum period is suggestive of ketonemia, insulin resistance, and catabolism of fat reserves (22). A target BCS of approximately 3.0 to 3.25 on a 5-point scale around calving normally maximizes milk production and decreases the risk of infectious and metabolic diseases in dairy cattle. A BCS loss of 0.5 to 1 point during the postpartum period is commonly observed. Losses of more than 1 point are considered alarming, are associated with impaired metabolic and reproductive performance, and should, therefore, be avoided (23). In our study, changes in BCS in both groups from the prepartum to the postpartum period reflected the stable mobilization of body reserves during lactation, which may have supported buffalo productivity.

In the context of milk production, buffaloes in Group I receiving transition feed supplementation produced a significantly higher milk yield (8.35 ± 0.18 vs. 6.25 ± 0.20 kg/day). This indicates that transition feed supplementation enabled buffaloes to sustain a better BCS without compromising lactation yield, suggesting more efficient nutrient partitioning. Our findings are in agreement with those of Jabbar et al. (24), who reported that milk yield increased significantly (*p* < 0.05) with the inclusion of cottonseed cake in the concentrate diet fed to cows. The role of critical micronutrients supplemented during the peripartum period in buffaloes was discussed by Jyani et al. (25), who observed that vitamin E and selenium act as cellular antioxidants that prevent oxidative injury to cell membranes and thus serve as important elements in the body’s defense against oxidative stress. In addition, minerals such as zinc and manganese have been reported to improve DM intake in peripartum cattle. The antioxidant effects and improved DM intake may have contributed to improved milk production performance in buffaloes. The role of methionine in enhancing milk production in dairy cattle has also been discussed by Wei et al. (26), who reported increases in energy-corrected milk, milk protein concentration, and milk protein yield. Methionine also enhanced cell proliferation and β-casein synthesis in bovine mammary epithelial cells (BMECs). This may be due to the uptake of peptides by the mammary glands for utilization in milk protein synthesis. Methionine supplementation increased the expression of peptide transporter 2 (PepT2) and the concentrations of gluconeogenic amino acids, thereby improving lactation performance. Strategic supplementation with microminerals such as zinc, magnesium, and copper has also been reported to affect milk parameters, including pH, somatic cell count, and CMT results. A recent study showed significant decreases in CMT results, somatic cell counts, and milk pH in supplemented buffaloes (27). The lower somatic cell counts and CMT results can be attributed to cell-mediated immune responsiveness and zinc-mediated keratin formation in the lining of the teat canal, which may entrap bacteria and prevent the upward movement of microorganisms into the mammary gland (28). The practical significance of the transition feed fed to buffaloes is reflected in the substantial increase in milk yield (2.35 kg/day), which can considerably enhance overall farm milk production and potentially offset part of the cost of transition feed supplementation while supporting improved metabolic adaptation during the transition period.

In addition to energy homeostasis, calcium homeostasis is another critical aspect of transition period management. During the peripartum period, urine pH has been considered an important indicator of calcium homeostasis. Our study underscores the pivotal role of anionic salts used in transition feed in maintaining urine pH in buffaloes. Similar observations were reported by Seifi et al. (29), who demonstrated the effects of anionic salts in preventing peripartum diseases. Urine pH analysis is used to assess the degree of metabolic acidosis. The target urine pH during the peripartum period in dairy cattle is approximately 6–6.8 (30) to maintain calcium homeostasis. Ruminants fed forage-based diets have high potassium concentrations, and a state of compensated metabolic alkalosis may occur. This metabolic alkalosis is further exacerbated during the peripartum period and reduces the ability to maintain calcium homeostasis, thereby limiting the synthesis of vitamin D. Appropriate feeding during the transition period with anion-rich diets can be an effective strategy to prevent hypocalcemia and maintain blood pH (30, 31). Mild metabolic acidosis affects parathyroid hormone receptors in the kidneys and bone and may enhance calcium reabsorption from the kidneys and calcium mobilization from bone, thereby increasing circulating calcium concentrations. These findings support the strategy of feeding transition feed during the peripartum period to help prevent production diseases, particularly milk fever in dairy cattle and buffaloes.

Moreover, our findings highlight the potential of transition feed supplementation to maintain major hematologic parameters in buffaloes, including WBCs, lymphocytes, and RBCs, during the transition period. Similarly, Sadeghian et al. (32) observed increased lymphocyte proliferation upon supplementation of vitamin E and selenium in the ration of dairy cattle. Selenium is considered an important factor in lymphocyte proliferation and transferrin receptor expression (33). Meglia et al. (34) conducted a study in HF cows during the transition period using a ration consisting of grass silage, concentrates, and hay. Total WBC and RBC counts and hemoglobin concentrations were significantly higher (*p* < 0.05) at calving than before and after calving in the feeding group compared with the control group. As dairy cattle approach calving, there is a substantial decrease in glucose concentrations, which may reflect increased fetal glucose utilization (35). This decrease has also been attributed to increased lactose synthesis during early lactation and insulin resistance in peripartum cattle (36, 37). In addition, DM intake by peripartum buffaloes may decrease by 10–30% because of increased fetal growth, decreased ruminal volume, and related factors (6). The transition feed fed to buffaloes had lower bulk density and was readily accepted, thereby helping to maintain daily DM intake. Considering its DM, organic matter, protein, and other nutrient contents, the transition feed provides the important nutritional components, including energy, protein, trace minerals, and vitamins, needed to support adequate production and reproductive function without compromising feed intake.

Components such as soyabean meal and cottonseed cake are metabolized into polyamines and arginine, which are considered important factors in stimulating erythropoiesis and hematopoiesis. Arginine and amino acids play pivotal roles in lymphocyte proliferation and immune boosting (38). Regarding minerals, magnesium plays a role in erythropoietin responsiveness, which is important for the survival, proliferation, and differentiation of erythrocytes in the bone marrow (39). Therefore, Mg^2+^ deficiency may affect erythropoiesis by disrupting the NF-κB (nuclear factor κB) pathway, indirectly altering cellular homeostasis, triggering changes in the erythrocyte membrane, and accelerating RBC aging (40). In addition, zinc plays a pivotal role in the optimal function of growth hormone (GH) and IGF-1, which act together with erythropoietin (a glycoprotein) to support hematopoiesis (41). Copper is also an important component of the enzyme ceruloplasmin, which is involved in iron metabolism and the incorporation of iron into hemoglobin. The feed components encompass the essential elements required for erythropoiesis during the peripartum period, particularly to support fetal development. The transition feed can be included as a supplement to the basal diet to help prevent anemia-like conditions and exert immunomodulatory effects that may support immune cell function and resistance to infection around calving.

The transition period imposes a substantial metabolic load on the liver, which serves as the central organ regulating gluconeogenesis, lipid transport, and protein synthesis. In this study, buffaloes fed the transition feed showed lower activities of SGOT and GGT and higher levels of total protein, albumin, and cholesterol than those in the control group. These findings collectively suggest improved hepatic function and reduced hepatic stress. Albumin is considered a negative acute-phase protein, and its level may decrease during the transition period due to increased inflammatory responses, amino acid catabolism, and reduced protein reserves associated with decreased DM intake (42). The transition feed comprised meals and cakes that provided a rich source of protein to the buffaloes, potentially limiting the mobilization of body protein to meet metabolic demands. This would allow protein to be utilized for fetal growth, immune function, and hematopoiesis during the peripartum period without compromising the protein status of the dam. Similarly, lower BUN concentrations may reflect reduced protein catabolism, while higher cholesterol concentrations may reflect improved lipoprotein synthesis (43, 44). Collectively, the biochemical profile suggests that transition feed supported normal hepatic function and may have reduced the mobilization of body reserves during early lactation.

Calcium homeostasis represents one of the physiological challenges immediately after parturition owing to the sudden demand for calcium for colostrum production and subsequent milk synthesis. The transition feed contained vitamin D3, anionic salts, and essential minerals designed to support calcium absorption and mobilization. The buffaloes receiving transition feed maintained higher plasma calcium levels than the control group. A negative dietary cation–anion difference helps prevent milk fever and can be achieved by increasing the proportion of anionic salts (e.g., chloride and sulfur) and reducing the proportion of cations (e.g., sodium and potassium) in the feed (45). Furthermore, magnesium plays a role in parathyroid hormone function and the regulation of plasma calcium concentrations (46). This inclusion of vitamin D3 and magnesium in the transition feed may therefore have helped maintain plasma calcium concentrations and reduce the risk of hypocalcemia (milk fever) in buffaloes during the immediate postpartum period.

Owing to reduced DM intake in the last few weeks of gestation, dairy cattle are likely to enter an NEB, accompanied by insulin resistance and growth hormone-induced lipolysis. As an adaptive response, lipid and protein reserves are mobilized, thereby increasing the flux of NEFAs to the liver. This leads to the accumulation of triglycerides in the liver, causing hepatic steatosis (fatty liver). In the later stage, hepatic cells may have a reduced capacity to use NEFA as fuel, thereby increasing the production of β-hydroxybutyrate and acetoacetate and contributing to a ketotic state (45). In this study, significantly lower BHBA concentrations were observed in Group I than in Group II throughout the transition period, indicating a more favorable energy balance in transition feed-supplemented buffaloes. Although BHBA concentrations increased progressively from the prepartum to postpartum period, the magnitude of this change was substantially smaller in the supplemented buffaloes. The significant Group × Time interaction further indicates a differential time course of BHBA between the groups, with supplementation attenuating the marked postpartum increase relative to the unsupplemented group. The divergence between the groups around calving and during the early postpartum period may indicate better availability and utilization of energy, thereby reducing fat mobilization and ketogenesis. Kour et al. (47) similarly reported an increase in BHBA concentrations from the prepartum to postpartum period. Free fatty acids, NEFA, and BHBA are considered potential indicators of NEB and ketosis in dairy animals, in addition to blood glucose levels (48). High BHBA concentrations may induce oxidative stress in hepatic tissues, inflammation, and apoptosis (49). Yang et al. (77) reported that oxidative stress-triggered pathways during NEB may serve as central regulators of pro-inflammatory cytokine production in tissues such as hepatic and mammary epithelial cells. Oxidative stress markers, particularly MDA (malondialdehyde) and LPO (lipid peroxidation), are elevated during the transition period due to increased levels of BHBA and NEFA. Concurrently, the antioxidant defense mechanism is compromised, with reduced levels of superoxide dismutase, catalase, and glutathione peroxidase. From an immunologic point of view, TNF-α and IFN-γ are considered pro-inflammatory mediators that act as important activators of macrophages against infectious pathogens (50). During the transition period, BHBA and NEFA may enhance the expression of these cytokines in peripheral mononuclear cells, thereby impairing immune regulation by peripheral leukocytes (51). Elevated cytokine levels during the transition period indicate an inflammatory response and may reflect increased susceptibility to infection in buffaloes. In our study, the inflammatory cytokine profile indicated that TNF-α concentrations were consistently lower in Group I than in Group II across the transition period, with both groups showing a marked increase from −30 days to the day of calving, followed by lower levels during the postpartum period. This pattern suggests that transition feed supplementation may have modulated the immune response associated with the transition period. In contrast, IFN-γ concentrations were significantly lower in the transition feed-supplemented group (Group I) from −30 to −15 days, while no significant differences were observed between the groups from the day of calving through +30 days postpartum. The significant Group × Time interactions for both cytokines indicated that their temporal patterns varied by stage of the transition period and supplementation. These outcomes may imply improved immunometabolic adaptation to the physiological changes of late gestation and lactation, potentially contributing to better defense against infectious and inflammatory stressors during this period. Transition feed supplementation in buffaloes resulted in lower BHBA and pro-inflammatory cytokine levels and reduced lipid peroxidation, suggesting a more favorable energy balance, improved antioxidant defense, potentially reduced hepatic lipid accumulation, and a potentially lower risk of metabolic and inflammatory disorders. A study by Reist et al. (52) reported that an increased concentrate level of feeding, up to 50% of total DM, reduced the levels of BHBA and NEFA. Tienken et al. (53) observed that cows provided with a higher concentrate level in the basal ration consumed more DM, had higher energy intakes, and had a more positive calculated energy balance during the prepartum period and, consequently, had lower levels of NEFA and BHBA postpartum. In the study by Wang et al. (54), supplementation with soybean protein in addition to the basal diet decreased the levels of pro-inflammatory mediators (*p* < 0.05) compared with the non-supplemented group. Overall, these findings highlight the potential role of balanced transition feed in supporting nutritionally adequate feeding strategies for buffaloes. Such strategies may help improve milk production, udder health, BCS, and immune modulation.

With appropriate amounts of vitamins (E, A, and D) and essential minerals (copper, zinc, calcium, etc.) present in the transition feed, oxidative stress parameters showed significant Group, Time, and Group × Time effects for LPO, GPx, catalase, and SOD. The antioxidant enzymes GPx and catalase showed increased activities in Group I, particularly during the prepartum and early postpartum periods, signifying improved antioxidant defense. As lactation progressed, the activities of both enzymes decreased; however, GPx showed partial recovery by +30 days, whereas catalase maintained relatively higher activity. The significant Group × Time interaction for LPO indicates that the time-course pattern of lipid peroxidation may reflect a modulatory effect of transition feed on oxidative stress status. When integrated with the enzymatic antioxidant profiles, these findings support the potential contribution of nutritional supplementation to maintaining oxidative homeostasis during this period. Similar findings were reported by Dimri et al. (55), who observed a significant reduction in lipid peroxidation (*p* < 0.05) during the transition period in water buffaloes and increased superoxide dismutase activity in the vitamin- and mineral-supplemented group. Antioxidants prevent oxidative damage to target cells and are categorized into non-enzymatic forms (tocopherols and ascorbic acid) and enzymatic forms (SOD, catalase, and GPx). Superoxide dismutase is a copper- and zinc-dependent enzyme that catalyzes the dismutation of superoxide radicals to hydrogen peroxide and acts as the first line of defense against reactive oxygen species (56). Selenium plays a role in leukocyte migration and microbial killing through its contribution to GPx and thioredoxin reductase activity. Selenium supplementation during the transition period may reduce reactive oxidative species and enhance microbial killing (57). Similarly, zinc is crucial for neutrophil chemotaxis, maturation, and phagocytosis (58). Copper also plays a role in the functioning of superoxide dismutase and ceruloplasmin, which help protect cells from oxidative damage (59). Non-enzymatic antioxidants, particularly vitamin E, are important contributors to the antioxidant capacity of plasma. Vitamin E, at higher doses, may enhance the expression of SIRT1 (a sirtuin family protein) and Nrf2 (an essential transcription factor), suggesting a potential role in counteracting age-related cellular changes. SIRT1 is considered essential for DNA repair, inflammation, and the aging process. Nrf2 is instrumental in maintaining redox balance, and it promotes the synthesis of enzymes such as SOD, catalase, and GPx. A study reported that exogenous administration of vitamin E restored reduced RBC levels, and vitamin E is considered a lipophilic chain-breaking antioxidant that interrupts lipid peroxidation reactions, thereby reducing oxidative stress (60). LeBlanc et al. (61) observed a significant reduction in infections during the early postpartum period with supplementation of retinol during the late prepartum period. Supplementation of transition feed with essential trace minerals and vitamins may enhance antioxidant defense mechanisms and reduce cellular damage induced by reactive oxygen species.

The estimation of mRNA expression of molecular markers specific to production diseases has been a pivotal breakthrough in the diagnostic domain. The analysis of gene expression levels can help predict the occurrence of production diseases in advance; therefore, early nutritional/therapeutic interventions can be performed. Commonly, hypocalcemia and NEB are observed in dairy cattle soon after calving. Gene expression profiling in dairy cows with experimental hypocalcemia has been performed by Sasaki et al. (62), but limited microarray data in buffaloes have been documented. Our study has shed light on the expression of putative markers of hypocalcemia, NEB, and inflammation in buffaloes receiving transition feed supplementation. NUAK1 gene is a serine/threonine kinase that belongs to the AMPK-related family and is upregulated in milk fever/hypocalcemic conditions. These results showed that supplementation with transition feed reduced the probability of milk fever by downregulating the expression of the NUAK1 gene. In a study by Inazuka et al. (63), NUAK1 was found to play a role in the suppression of glucose uptake via negative regulation of the insulin pathway in rats. It has been reported that dairy cattle with hypocalcemia showed increased plasma glucose concentrations associated with reduced insulin secretion (64). Furthermore, proper regulation of glucose uptake is required for the proper functioning of immune cells, including lymphocytes. With upregulation of NUAK1, suppression of glucose uptake by immune cells is observed (65). To date, limited information is available on the role of NUAK1 in peripheral blood mononuclear cells in relation to insulin and glucose regulation. Further research is required to determine the physiological basis for upregulated NUAK1 mRNA expression in the PBMCs of dairy animals with hypocalcemia/milk fever. Another marker of hypocalcemia, NESP55, was also studied and showed lower expression in the transition feed group than in the control group. The GNAS cluster, which is a complex imprinted locus producing multiple protein-coding transcripts from different promoters, including NESP and GNAS, encodes NESP55 and Gsα protein, respectively. Reduced expression of the GNAS cluster, particularly NESP55, causes resistance to parathyroid hormone, leading to hypoparathyroidism (66). It has been well established that parathyroid hormone plays a role in calcium homeostasis. The lower NESP55 expression in the control group suggests lower calcium levels than in the transition feed group. This might be due to reduced parathyroid hormone effectiveness, leading to low calcium concentrations in buffaloes. Nutritional intervention with transition feed may, therefore, help prevent hypocalcemia in buffaloes during the immediate postpartum period.

In the context of NEB in buffaloes, CPT1A showed upregulation, and IGF-1 showed downregulation in the transition feed group. Similar findings were reported in a study by Loor et al. (67), which compared two groups of cattle fed ad libitum and a feed-restricted diet. The feed-restricted group showed greater upregulation of CPT1A and ADIPOR2, gluconeogenesis-related PC, and cholesterol synthesis-related SC4MOL genes, indicating greater hepatic oxidation of free fatty acids and, thus, greater metabolic stress on the liver. Douglas et al. (68) conducted a study of moderate grain-fat supplementation at ad libitum or restricted levels in cows during the 21 days preceding calving. They observed higher CPT (carnitine palmitoyl transferase) activity in the liver mitochondria of cows previously fed a restricted diet. CPT activity declined more rapidly in cows fed ad libitum during the prepartum period, indicating more stable hepatic metabolism and less metabolic stress during the postpartum period. CPT1A plays a role in hepatic fatty acid β-oxidation and energy metabolism. This involvement of CPT1A explains its upregulation during this phase, with increased β-oxidation of fatty acids and lipid metabolism (47). The greater upregulation of CPT1A in the control group suggests increased fatty acid mobilization and oxidation, as well as ketone body formation, potentially resulting in hepatic metabolic overload. Therefore, appropriate inclusion of essential components in the transition feed may help alleviate this hepatic overload, reduce ketone body formation, and prevent NEB. Nutrition plays an important role in the regulation of GH secretion and plays a pivotal role in the partitioning of nutrients and the metabolism of carbohydrates and lipids (69). Decreased GHR1 mRNA expression in the liver during early lactation leads to a decrease in growth hormone-dependent IGF-1 synthesis (70). During the periparturient period, dairy cattle undergo several metabolic changes to support lactation, which can lead to protein and carbohydrate undernutrition. This causes a marked reduction in GHR expression and a subsequent decrease in IGF-1 synthesis (71).

Adipose tissues are considered sources of several pro-inflammatory cytokines, such as interleukins and tumor necrosis factor-α. The enhanced expression of these cytokines can induce a pro-inflammatory environment and facilitate oxidative damage, including increased free radical production (72). Increased hepatic triglyceride accumulation (fatty liver) and the abundance of metabolic genes related to fatty acid uptake and storage have been observed following administration of recombinant bovine TNF-α during late pregnancy in cattle. Conversely, decreased expression of gene transcripts associated with processes such as gluconeogenesis is suggestive of decreased glucose production due to inflammatory pathways (54). In the study by Kokou et al. (73), there was an increase in the level of anti-inflammatory mediators in cattle fed graded levels of soybean concentrate. Galyean et al. (72) reported that cattle supplemented with copper and zinc during pregnancy had lower plasma TNF-α levels than those fed a basal diet. Wang et al. (54) conducted a trial to investigate the effect of replacing the protein source with soybean protein and observed that the mRNA levels of anti-inflammatory cytokines (TGF-β, IL-10, epinecidin, MHCIIβ, and hepcidin) were upregulated, whereas those of pro-inflammatory cytokines were downregulated (TNF-α, IL-10, INF-γ, and IL-12P40). These findings support the potential role of nutritional management in buffaloes during the transition period in mitigating inflammatory cascades and the associated mediators of infectious and production diseases.

## 5 Conclusion

The nutritional intervention in buffaloes during the transition period resulted in significant improvements in the levels of WBCs, granulocytes, RBCs, Hb, total protein, albumin, glucose, calcium, and sodium in the transition feed group. In contrast, decreased levels of SGOT, GGT, BUN, and lipid peroxidation were observed. The transition feed group had lower levels of TNF-α and INF-γ, as determined by both ELISA and qPCR, suggesting an immunomodulatory effect of the feed. Overall, the altered expression of disease-specific genes highlights the role of targeted nutritional supplementation in mitigating the occurrence of production and infectious diseases during the transition period, thereby supporting improved milk production and overall health of buffaloes.

## Data Availability

The original contributions presented in the study are included in the article/Supplementary material, further inquiries can be directed to the corresponding authors.
