# Supplementary material for: Development and biomarker-based validation of a novel feed formulation for managing production diseases in dairy buffaloes
Source: Front Vet Sci. 2026 Sep 8;13:1937866. doi: 10.3389/fvets.2026.1937866 (PMC13595057; doi:10.3389/fvets.2026.1937866)
Supplement: Supplementary file 1 [file Table_1.DOCX]

**Supplementary Table 1: Primer sequences and annealing temperatures of genes of interest**

|  | **Genes** | **Annealing temperature** | **Size** | **Sequence** |
| --- | --- | --- | --- | --- |
| **Hypocalcemia** | NUAKI (Upregulated) | 58.3 ºC | 202 bp | F: TGAAAATGCCAAGTGCTCTC  R: AGGAATGATACCATGCCAGT |
|  | NESP55 (Downregulated | 62.3 ºC | 116bp | F: TTTTTAATGCTGCACAACACGAT  R: GCCACAAATGTTCCTTCTCTCTTT |
| **Negative Energy Balance** | CPT1A (Upregulated) | 58.7 ºC | 292bp | F: TTATGTGAGCGACTGGTGGG  R: GTGCTGGATGGTGTCTGTCT |
|  | IGF1 (Downregulated | 59.6 ºC | 140bp | F:CATCCTCCTCGCATCTCTTC  R: GAAATAAAAGCCCCTGTCTCC |
| **Cytokines** | TNF-alpha  (Upregulated) | 58.9 ºC | 272bp | F: TAACAAGCCGGTAGCCCACG  R:TCTTGATGGCAGACAGGATG |
|  | INF-gamma  (Upregulated) | 58.7 ºC | 98bp | F:TGGAGGACTTCAAAAAGCTGATT  R:TTTATGGCTTTGCGCTGGAT |

**Supplementary Table 2: qPCR cyclic condition**

| **Steps** | **Temperature** | **Cycles** | **Time** |
| --- | --- | --- | --- |
| Pre incubation | 95 ºC | 1 | 5min |
| Amplification  (3 steps) | 95 ºC | 40 | 20 sec |
|  | Annealing temperature of the gene of interest | 40 | 15 sec |
|  | 72 ºC | 40 | 15 sec |
| Pre melt | 95 ºC | 1 | 5 sec |
| Melting | 70 ºC | 1 | 1 min |
| Melting | 95 ºC | 1 | 40 sec |
| Cooling | 40 ºC | 1 | 30 sec |
